# Identification of a Subpopulation of Astrocyte Progenitor Cells in the Neonatal Subventricular Zone: Evidence that Migration is Regulated by Glutamate Signaling

**DOI:** 10.1007/s11064-024-04326-2

**Published:** 2025-01-09

**Authors:** Zila Martinez-Lozada, Alain M. Guillem, Isabella Song, Michael V. Gonzalez, Hajime Takano, Esha Parikh, Jeffrey D. Rothstein, Mary E. Putt, Michael B. Robinson

**Affiliations:** 1 Departments of Pediatrics and Systems Pharmacology & Translational Therapeutics, The Children’s Hospital of Philadelphia, University of Pennsylvania, Philadelphia, PA 19104-4318 USA; 2 Department of Neuroscience, College of Psychology and Neuroscience, Nova Southeastern University, Fort Lauderdale, FL 33328 USA; 3 Center for Cytokine Storm Treatment & Laboratory, Perelman School of Medicine, University of Pennsylvania, Philadelphia, PA 19104 USA; 4 Department of Neurology, University of Pennsylvania, Philadelphia, PA 19104 USA; 5 Department of Neurology, Johns Hopkins University School of Medicine, Baltimore, MD 21205 USA; 6 Department of Biostatistics, Epidemiology & Informatics, Perelman School of Medicine, University of Pennsylvania, Philadelphia, PA 19104 USA

**Keywords:** Astrocytes, Development, Astrocyte precursor cells, Migration, Glutamate receptor, And glutamate transport

## Abstract

In mice engineered to express enhanced green fluorescent protein (eGFP) under the control of the entire glutamate transporter 1 (GLT1) gene, eGFP is found in all ‘adult’ cortical astrocytes. However, when 8.3 kilobases of the human GLT1/EAAT2 promoter is used to control expression of tdTomato (tdT), tdT is only found in a subpopulation of these eGFP-expressing astrocytes. The eGFP mice have been used to define mechanisms of transcriptional regulation using astrocytes cultured from cortex of 1–3 day old mice. Using the same cultures, we were never able to induce tdT^+^ expression. We hypothesized that these cells might not have migrated into the cortex by this age. In this study, we characterized the ontogeny of tdT^+^ cells, performed single-cell RNA sequencing (scRNA-seq), and tracked their migration in organotypic slice cultures. At postnatal day (PND) 1, tdT^+^ cells were observed in the subventricular zone and striatum but not in the cortex, and they did not express eGFP. At PND7, tdT^+^ cells begin to appear in the cortex with their numbers increasing with age. At PND1, scRNA-seq demonstrates that the tdT^+^ cells are molecularly heterogeneous, with a subpopulation expressing astrocytic markers, subsequently validated with immunofluorescence. In organotypic slices, tdT^+^ cells migrate into the cortex, and after 7 days they express GLT1, NF1A, and eGFP. An ionotropic glutamate receptor (iGluR) antagonist reduced by 50% the distance tdT^+^ cells migrate from the subventricular zone into the cortex. The pan-glutamate transport inhibitor, TFB-TBOA, increased, by sixfold, the number of tdT^+^ cells in the cortex. In conclusion, although tdT is expressed by non-glial cells at PND1, it is also expressed by glial progenitors that migrate into the cortex postnatally. Using this fluorescent labeling, we provide novel evidence that glutamate signaling contributes to the control of glial precursor migration.

**Supplementary Information:**

The online version contains supplementary material available at 10.1007/s11064-024-04326-2.

## Introduction

The glutamate transporter, called GLT1 (or excitatory amino acid transporter 2, EAAT2), is one of the most abundant proteins in the brain, and its expression is almost exclusive to astrocytes in the adult nervous system [1–5]. The presence of GLT1 at the astrocyte plasma membrane is critical for maintaining low extracellular glutamate and buffering/clearing synaptically released glutamate. In addition, we and others have shown that GLT1 can act as a signal transducing molecule (Reviewed in [6]). It is the only glutamate transporter that, upon deletion, causes death in mice [7]. Decreased expression of this transporter is observed in animal models of neurologic disease and *in postmortem* tissue of patients with neurodegenerative/neurologic diseases [8–10]. These observations prompted several groups to study the molecular mechanisms that regulate expression of this transporter. As part of this effort, one of our groups (J.D.R) developed a mouse line that uses the entire GLT1 (Slc1a2) mouse gene from a bacterial artificial chromosome to control expression of enhanced green fluorescent protein (eGFP)(BAC-GLT1-eGFP) [11]. Another transgenic mouse line that utilizes 8.3 kilobases of the proximal Slc1a2 human promoter to control expression of tdTomato (tdT) was also generated and has been used to study astrocyte heterogeneity and their morphological maturation [12–15]. In mice that carry both transgenes, (BAC-GLT1-eGFP/8.3-EAAT2-tdT) tdT is only observed in a subpopulation (~ 28%) of cortical astrocytes enriched in layer V, and always overlaps with eGFP in the cortex [12]. These double-positive astrocytes were found to be molecularly (both RNA and protein) and functionally distinct from astrocytes that only express eGFP, but they express comparable levels of GLT1 mRNA and protein [12]. We have used astrocytes prepared from cortex of young mice, postnatal day 1–3 (PND 1–3), to study transcriptional regulation of GLT1 and/or eGFP; expression of both proteins increases with several stimuli, including neurons and endothelia [16–21]. Three different members of the Robinson laboratory (Elizabeth Krizman, Meredith L. Lee, and Z.M.L.) have used cortical astrocytes from the 8.3-EAAT2-tdT PND 1–3 mice in attempts to induce tdT expression, but tdT was never observed.

During normal cortical development, astrocytes are generated directly from radial glial cells in the ventricular zone [22–25], bipotential glial progenitor cells in the subventricular zone (SVZ) [26–28], subpallial progenitor cells [29, 30] and NG2^+^ cells [31–34]. In most cases, these progenitors migrate into the cortex during late embryogenesis, prior to birth, but Goldman’s group and others had shown that some glial progenitor cells migrate from the SVZ into cortex after birth [26–28]. Based on this, we wondered if precursors for tdT expressing astrocytes might migrate into the cortex after birth, precluding our ability to induce expression in cultured astrocytes prepared from cortices of young mice. To address this question, we used the transgenic mouse lines, BAC-GLT1-eGFP and 8.3-EAAT2-tdT, to characterize the ontogeny of tdT^+^ cells. We also performed scRNA sequencing on eGFP and tdT expressing cells at PND1. We used organotypic slice cultures to study the migration of both cell types and performed explants of SVZ from reporter mice into slices prepared from wild-type mice to demonstrate that tdT-expressing cells from PND1-2 mice migrate into the cortex postnatally.

Because the molecular signals that direct the migration of glial progenitors are understudied, in part, due to the lack of specific markers for these cells [35], we took advantage of the expression of reporter proteins, tdT and eGFP, to investigate the cues that drive their migration. Prior studies have demonstrated that glutamate regulates the migration of cerebellar granule cells [36], cortical neurons [37–40], GABAergic interneurons [41], and oligodendrocyte precursor cells. Therefore, in this study, we also examined the effects of antagonists of ionotropic glutamate receptors (iGluR) or a pan-inhibitor of glutamate transport on the migration of these cells. These studies support earlier evidence indicating that some astrocyte precursors migrate into the cortex postnatally and provide the first evidence that migration of these cells is regulated by glutamate signaling.

## Materials and Methods

### Animals

A colony of double transgenic (BAC-GLT1-eGFP/8.3-EAAT2-tdTomato, from now on referred to as eGFP/tdT) reporter mice [12], and a colony of C57BL/6 J wild-type mice (Jackson Laboratory, RRID: IMSR_JAX:000664) were maintained at the animal facility of The Children’s Hospital of Philadelphia. Tail snips were collected, and genotyping was performed by RT-PCR with the following primers for eGFP forward CTACGCCAAGCTGACCCTGA reverse CGA TGT TGT GGC GGA TCT and for 8.3 kb EAAT2 forward AGACACATATCTTTACTCCTGCCT reverse TAGCACCTGGACACAGTCTC. When the brains were harvested from neonates, the expression of reporter proteins was examined using an epifluorescence microscope equipped with a 4X objective to identify the genotype of the animal. Male double reporter mice were crossed with C57BL/6 J wild-type female mice (maximum of four pregnancies/mouse). The double reporter positive (eGFP^+^/tdT^+^) pups of these crossings were used for most of the experiments unless stated otherwise, and double reporter negative (eGFP^−^/tdT^−^) littermates were used as controls or to provide wild-type tissue for explant cultures. Up to five mice were housed per cage in standard controlled temperature, humidity, and light conditions and had ad libitum access to food and water. This mouse colony was monitored twice per week for any evidence of pain or distress. We consulted with the attending veterinarian on the rare occasion of an issue. Mice were either medically treated or euthanized to minimize pain and distress. All studies were approved by the Institutional Animal Care and Use Committee of The Children’s Hospital of Philadelphia and followed the National Institutes of Health Guidelines for the Care and Use of Laboratory Animals.

### Sample Preparation, Single-Cell RNA Sequencing (scRNA-seq), and Data Processing

After the mice were genotyped as described above, the dorsal-anterior forebrain (Fig. 3a) from three double-positive (eGFP^+^/tdT^+^) P1 mouse brains were dissected, cut into small pieces, and incubated 25 min at 37 °C with a 2 mg/mL papain solution (Worthington Biochemical Corporation, Cat#LK003178) in hibernate E minus calcium media (BrainBits, Cat#HECA) swirling every 5 min. This was followed by mechanical dissociation with a Pasteur pipette, followed by 1000 µL and 200 µL pipette tips (20 passes through each). Cells were incubated for 15 min at room temperature with DRAQ7 (1:500, BioLegend, Cat#424,001) in sorting buffer (PBS 1X + fetal bovine serum 4%). Fluorescence-activated cell sorting (FACS) was performed in the Flow Cytometry Core Laboratory at The Children’s Hospital of Philadelphia using an electrostatic droplet FACSJazz sorter with Isoflow sheath fluid (Beckman Coulter, Cat#8547008) and a 100 µm nozzle. Debris and aggregates were excluded using the pulse width of the triggering parameter and dead cells based on DRAQ7 staining (using the red laser Ex 640 nm, Em 660/20 nm). Then, we gated cell populations based on eGFP fluorescence (using the blue laser Ex 488 nm, Em 530/40 nm) and tdT fluorescence (using the yellow-green laser Ex 561 nm, Em 585/29 nm). Unstained and single fluorophore-labeled cells were used to verify the gates. Cells were purified using a 1.0 Drop Pure sort mode and collected into sorting buffer (427,480 tdT^+^ and 260,473 eGFP^+^ cells were collected) and quickly isolated by centrifugation at 350×*g* for 5 min. A purity test with an aliquot of the sorted cells showed 99% purity for both sorted cell populations. Viability was 93% and 92% for tdT^+^ and eGFP^+^ cells, respectively.

Next-generation sequencing libraries were prepared by the Center for Applied Genomics at The Children’s Hospital of Philadelphia using the 10 × Genomics Chromium Single Cell 3’ Reagent kit v3 per manufacturer’s instructions. Libraries were uniquely indexed using the Chromium i7 Sample Index Kit. After preparing a library and performing quality control on a High Sensitivity D1000 ScreenTape station, 17,596 tdT^+^ and 16,956 eGFP^+^ cells were loaded and sequenced on an Illumina NovaSeq sequencer in a paired-end, single indexing run. Sequencing for each library targeted 20,000 mean reads per cell. Data were then processed using the cellranger pipeline (10 × genomics, v6.0.0) for demultiplexing and alignment of sequencing reads to the mm10 transcriptome, with the tdT (GenBank Accession #AY678269.1) and eGFP (GenBank Accession #MK387175.1) transcripts added to produce the feature-barcode matrices. The Seurat suite [42] of tools within the R compute environment was used for downstream QC and analysis. Briefly, cells expressing fewer than 200 and more than 5000 unique features and greater than 25% mitochondrial gene expression were excluded from downstream analysis. Seurat, in conjunction with Harmony [43] were used to perform data normalization, scaling, and cell clustering. The resulting clusters were annotated using canonical markers. Differentially expressed genes were determined using a Wilcoxon rank-sum test. A Bonferroni correction was used to account for multiple testing. To account for batch effects, normalization and scaling were performed on the aggregated dataset within Seurat. Briefly, raw gene expression counts for each cell were divided by its total count. After normalization, gene counts were scaled using a scaling factor of 10,000 and log-transforming the result. Further, the Harmony package, was used to integrate the samples and project cells into a shared embedding seen in Online Resource 3c.

### Immunofluorescence (IF)

Pups from the crossing of male double reporter (eGFP/tdT) mice with female wild type C57BL/6 J mice were decapitated at times indicated in each figure. Brains were dissected and genotyped with an epifluorescence microscope. Double reporter brains were submerged in 4% paraformaldehyde for 1 h to overnight (depending on the age of the animals) at 4 °C, cryoprotected in 30% sucrose, snap frozen, and stored at −80 °C. Tissue was cut using a Leica cryostat into 30−50 μm coronal or sagittal sections and placed directly into Superfrost Plus microscope slides (Fisherbrand Cat#22–037-246). Sections were rinsed twice with PBS. If no staining procedure was required, sections were mounted with Vectashield + DAPI (Vector Laboratories Cat#H-1200). Images were taken with a DMi8 SP8 Leica confocal microscope equipped with a 40X objective (Leica Microsystems, RRID: SCR_018169), and the sequential mode was used to avoid cross-contamination of fluorophores across channels.

If a staining procedure was required, sections were rinsed two times with PBS/Tween (0.05% Tween 20 in 1X PBS), then treated with blocking buffer (5% goat serum in 0.4% Triton X-100 in 1X PBS) for 1 h at room temperature. Primary antibodies diluted in blocking buffer were added to sections and incubated overnight at 4 °C. The primary antibodies used were rabbit anti-GLT1 (C-terminal-directed; 1:100, final concentration of 2.5 μg/mL; Cat#GLT-1a RRID: AB_2314565 [3],); mouse anti-GLAST (1:100; MACS Miltenyi Biotec Cat#130-095-822; RRID:AB_10829302); rabbit anti-BLBP (1:100; Millipore Sigma-Aldrich Cat#ZRB13190; RRID:AB_2920766); or rabbit anti-NFIA (1:500; Abcam Cat#ab228897; RRID:AB_2923081). The next day, sections were washed three times, five minutes each, with PBS/Tween. Secondary antibodies diluted 1:500 in blocking buffer were added to sections and incubated for 2 h at room temperature. The secondary antibodies used were Alexa Fluor 633 Goat anti-Rabbit (Thermo Fisher Scientific Cat# A-21071; RRID: AB_2535732) or Alexa Fluor 633 Goat anti-Mouse (Thermo Fisher Scientific Cat# A-21052; RRID: AB_2535719). Sections were rinsed three times with PBS/Tween, then mounted with Vectashield + DAPI (Vector Laboratories Cat#H-1200). Images were taken with a DMi8 SP8 Leica confocal microscope equipped with a 40X objective (Leica Microsystems, RRID: SCR_018169), and the sequential mode was used to avoid cross-contamination of fluorophores across channels. Images were taken in the cortex (CTX), subventricular zone (SVZ), ventricular zone (VZ), and striatum (STR) in anterior and medial brain slices. The percentage of cells with immunoreactivity for the different antibodies was quantified in 3–4 fields per animal, with three mice from different litters per antibody. Images were compiled using Fiji software (RRID: SCR_002285).

### Immunofluorescence (IF) Image Analysis

For membrane/cytoplasmic markers (GLT1, GLAST, and BLBP), the image segmentation software Cellpose (v2.2, RRID:SCR_021716) was used to segment the cells expressing eGFP or tdT reporter proteins. Cellpose’s built-in cyto2 model was further trained on 50% of the data and applied to the other 50% for automatic segmentation. ImageJ-FIJI (RRID:SCR_002285) was used to convert Cellpose’s segmentation output into a binary mask, and a custom MATLAB (RRID:SCR_001622) app was used to identify overlaps of the binary mask (eGFP or tdT cells) with the cytoplasmic antibody staining (BLBP). The MATLAB app can also generate donut-shaped cellular outlines based on the eGFP or tdT mask and was used to identify overlaps with the membrane staining (GLT1 and GLAST). A threshold of > 0.6 overlap was used to consider a cell as positive, and the percentage of cells that were double-positive was calculated using the number of eGFP^+^ or tdT^+^ cells as the denominator. For the nuclear markers (NFIA or Olig2), Cellpose was first used to segment nuclei in DAPI staining. ImageJ-FIJI was used to convert Cellpose’s segmentation output into a binary mask. If DAPI images were captured separately from other IF images, the Fiji plugin bUnwarpJ was used to align the binary mask with the IF images. The custom MATLAB GUI app was used to identify overlaps of the binary mask (DAPI) with reporter proteins (eGFP or tdT) or overlaps of the binary mask (DAPI) with NFIA or Olig2 staining. A threshold of > 0.6 area overlap was used to consider a cell as positive, and percent positive rates were calculated using the number of eGFP^+^ or tdT^+^ cells as the denominator.

### Organotypic Brain Cultures

We prepared acute brain slices from double reporter (eGFP^+^/tdT^+^) mice pups. Briefly, brains from postnatal day (PND) 1–2 mouse pups were dissected and placed in ice-cold Hank’s balanced salt solution (HBSS). Brains were genotyped using an epifluorescence microscope and then embedded in a solution of 4% low-melting agarose (Invitrogen, Cat#16520–100) in sterile artificial cerebrospinal fluid (aCSF) (KCl 5 mM, MgCl_2_(6H_2_0) 2 mM, CaCl_2_ 1 mM, sucrose 280 mM, glucose 20 mM, HEPES 10 mM, pH7.3). Using a vibratome (Leica Microsystems, model VT1000), brains were sliced into 300 µm thick coronal sections and collected in sterile ice-cold aCSF solution. The slices were placed in a glass bottom plate (Maltek corporation, P35GINV-0–20-C) containing 1 mL of media A (Neurobasal Invitrogen Cat# 21103-049, 25% Horse Serum Gibco Cat#2605-088, 25% HBSS Gibco Cat#14175-079, 2% Gem21 Neuroplex Gemini Cat#400-160, 100 µg/mL Pen/Strep Invitrogen Cat#15140122, 10 mM HEPES Gibco Cat#14175-079, 2 mM GlutaMAX Invitrogen Cat#25030081, and 36 mM glucose Fisher Cat#D16-500). After recovery for 14 to 18 h in an incubator (at 37 °C in 5% CO_2_), slices were imaged every 30 min on a DMi8 SP8 Leica confocal microscope equipped with a 40X objective (Leica Microsystems), using the 488 and 594 nm laser lines for eGFP and tdT, respectively, for up to 2 h. Slices were always placed in the same position on the microscope stage, with the cortex facing up. Between each imaging period the slices were returned to the incubator. Anatomic structures visualized with the bright field were used to orient the experimentalist and to facilitate the imaging of the same brain area. Cell migration was calculated by measuring the distance between the center of the implant site (IS) and the final position of the cell using the distance between two points formula (${\text{d}}\, = \,\surd \left( {\left( {{\text{x}}_{{2}} {-}{\text{x}}_{{1}} } \right)^{{2}} \, + \,\left( {{\text{y}}_{{2}} {-}{\text{y}}_{{1}} } \right)^{{2}} } \right)$). The rate of migration was calculated as the distance migrated divided by the duration of the experiment (v = d/t). All positions were determined using measurements with Fiji software (RRID: SCR_002285). Migration of five cells from 2–3 slices per mouse was measured in three independent experiments using mice from different litters and used to calculate the average rate of migration.

### Organotypic Cultures with Explants

Mice from the same litter were used for each experiment. Slices from double negative (WT, eGFP^−^/tdT^−^) mice were used as recipients, while single (eGFP^+^/tdT^−^ or eGFP^−^/tdT^+^) or double (eGFP^+^/tdT^+^) reporter mice were used to provide donor explants. When migration from explants of single reporters was compared, the same double-negative mouse was used to prepare recipient slices (each hemisphere used from one of the two reporters).

Brain slices (recipients and donors) were prepared as described above, but after sectioning, the recipient brain slices were placed on a 0.4 mm membrane insert (Millipore Cat#PICM0RG50) and positioned in 6-well plates containing 1 mL media A. A 1 mm diameter piece of tissue from the dorsolateral (DSL) SVZ or the striatum was micro-dissected using a sample corer (Fine Science Tools Cat#18035-50) under a dissection microscope. This tissue was implanted into the DSL-SVZ of the recipient slice. Brain slices were cultured for up to seven days at 37 °C in 5% CO_2_, with 1/3 of the media exchange every other day. NMDA receptor antagonists: 10 µM (5S,10R)-( +)−5-methyl-10,11-dihydro-5H-dibenzo[a,d]cycloheptene-5,10-imine maleate (( +)-MK 801 maleate, also known as dizocilpine) (Tocris Cat#0924/10, [44]) or 100 µM D-(-)−2-amino-5-phosphonopentanoic acid (D-AP5) (Tocris Cat#0106/1, [45, 46]), AMPA and KA receptor antagonists: 10 µM 2,3-dihydroxy-6-nitro-7-sulfamoylbenzo(f)quinoxaline disodium (NBQX disodium salt) (Tocris Cat#1044/1, [47, 48]) or 30 µM 6-cyano-7-nitroquinoxaline-2,3-dione disodium (CNQX disodium salt) (Tocris Cat#1045/1, [49–51]), or a pan blocker of glutamate transporters: 2 µM (3*S*)−3-[[3-[[4-(trifluoromethyl)benzoyl]amino] phenyl]-methoxy]-L-aspartic acid (TFB-TBOA) (Tocris Cat#2532/1; [52, 53]) were added to the media one hour after the implants were placed; these drugs were also included in the media used for feeding. Images of the implant site were taken after a recovery time (1 day post-implant) and/or after seven days in culture (7d post-implant) using a DMi8 Leica confocal microscope equipped with a 10 × objective (Leica Microsystems). Net cell migration was calculated by measuring the distance between the center of the implant site (IS) and the final position of the cell using the distance between two points formula $d=\sqrt{{\left({x}_{2}-{x}_{1} \right)}^{2 }+{\left({y}_{2}-{y}_{1} \right)}^{2}}$. Lateral cell migration was estimated as the distance the cells moved in the X axis ($d=\sqrt{{\left({x}_{2}-{x}_{1}\right)}^{2}}$) and the radial cell migration was estimated as the distance the cells moved in the Y-axis ($d=\sqrt{ {\left({y}_{2}-{y}_{1}\right)}^{2}})$. Using a custom MATLAB (RRID:SCR_001622) GUI app, we manually identified several points over the white matter tracts. This allows the app to identify a smooth line over the white matter tracts, then the app measures the shortest distance from the migrated cells to this line within the cortex (CC to CTX migration). All cells that moved in a Z-stack plane away from the IS (in all directions) were quantified (> 100 cells per condition). The positions (x,y-coordinates) of the migrated cells were determined using measurements with Fiji software (RRID: SCR_002285).

After seven days in culture, the slices were fixed in 4% PFA for 10 min at room temperature and washed three times with 1 × PBS, each for 10 min. Some sections were then processed for IF using anti-GLT1 or NFIA antibodies as described above, with the exception that primary antibodies were incubated for three nights at 4 °C, and the secondary antibodies were incubated overnight at 4 °C.

### Statistical Analyses

Figure legends list the numbers of independent experiments (n) that were conducted for the analysis using animals from different liters. A t-test was used to evaluate the difference in the expression of GLT1 or NFIA in the cortex versus the implant site seven days post-implant (Fig. 5).

Differences in the means of migration were assessed using mixed effects models to address correlations between repeated measurements of outcomes on animals from the same litter, and slices from the same animal. Litter and slice, nested within litter, were considered random effects. Outcomes of migration along the X-axis, Y-axis, net migration, and migration from CC to CTX were considered in separate models. The outcome variable was log-transformed to better approximate normality. The fixed effects in each model were the individual inhibitors. To reduce the number of comparisons and mitigate spurious false positives, we first compared the control to each of four families of inhibitors (D-AP5 and MK801 alone, CNQX and NBQX alone, the pairwise combination of agents in these two families, or TFB-TBOA) using an F-test. If the F-test was significant (p < 0.05), we compared individual inhibitors within the family to control using a Wald test.

The total number of cells that migrated or those that migrated from the CC to the CTX were similarly analyzed but summarized over the slices. Here litter was the sole random effect. Models were fit in R Version 4.4.1 using the lme4 and lmeTest packages [54, 55].

## Results

**Fig. 1:**
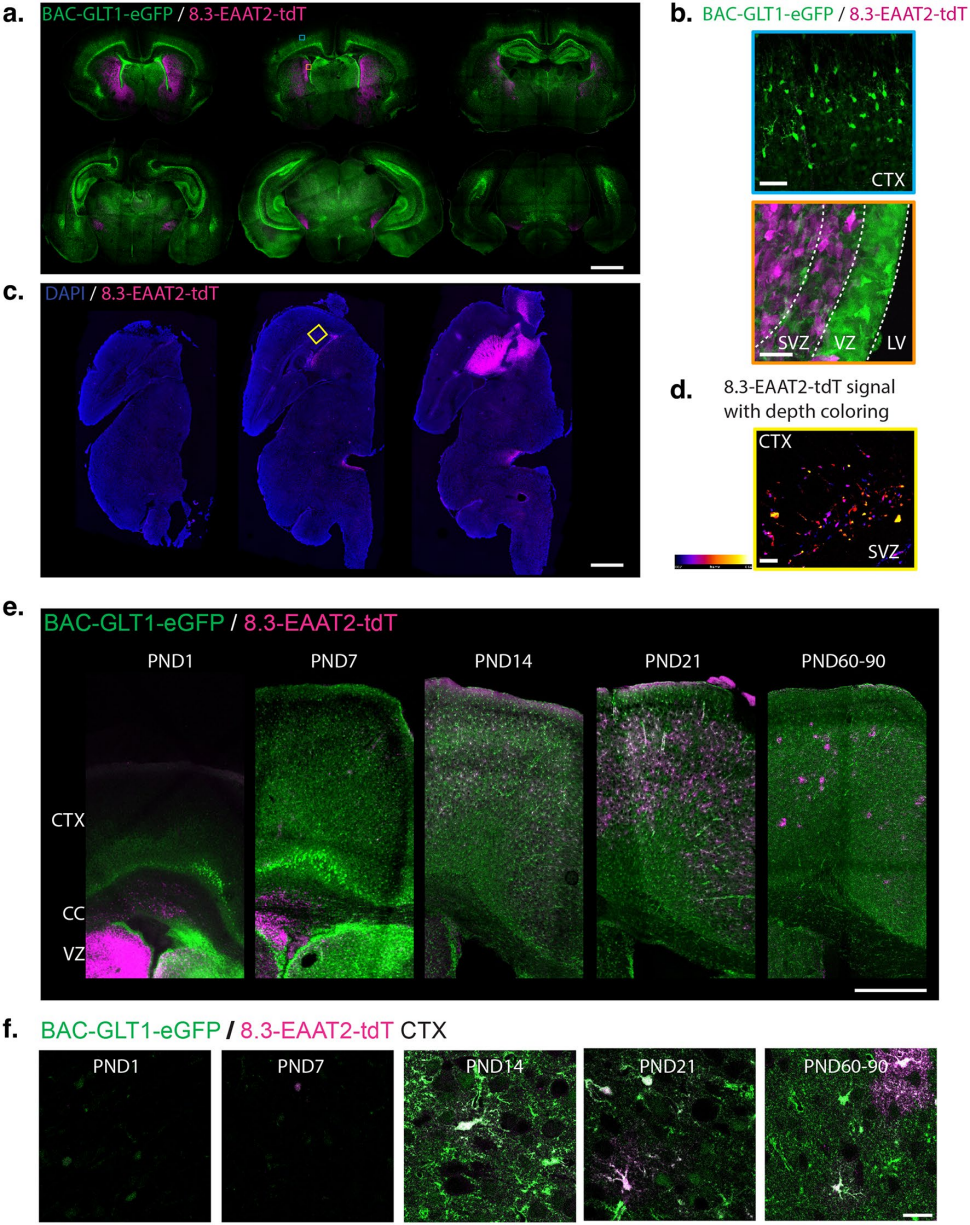
eGFP and tdT are expressed by different cells early in development. **a **& **b** Expression of tdTomato (tdT) and enhanced green fluorescent protein (eGFP) was analyzed in coronal brain sections prepared from double reporter BAC-GLT1-eGFP/8.3-EAAT2-tdT PND 1 mice. In panel **a** images are shown in rostral (top-left) to caudal (bottom-right) order. Scale bar = 1 mm. In panel **b** the images show the regions highlighted in the top, middle image of panel a: the top image from the cortex (CTX) in the blue box, and the bottom image from the subventricular zone (SVZ) highlighted in the orange box. Scale bars = 20 µm, VZ = ventricular zone, LV = lateral ventricle. **c **& **d** The localization of tdT was analyzed in sagittal sections prepared from single reporter 8.3-EAAT2-tdT postnatal day 1 mice. In panel **c** three different lateral to medial sections are shown. Scale bar = 1 mm. In panel **d** a z-stack of the SVZ region highlighted in the yellow box in the middle image of panel **c** is shown. The colors indicate different planes. **e**&**f** The localization of the reporter proteins tdT and eGFP was analyzed in the cortex of coronal brain sections prepared from BAC-GLT1-eGFP/8.3-EAAT2-tdT mice at the indicated developmental stages (PND 1, 7, 14, 21, and 60–90). Panel e, scale bar = 0.5 mm. Panel f shows higher magnification images in the cortex, all taken using the same laser settings to capture different expression levels of the reporter proteins. Scale bar = 25 µm. Three mice from a different litter were analyzed for each panel; representative images are shown

*Developmental time course of tdT and eGFP expression in mouse brain.* In adult double reporter BAC-GLT1-eGFP/8.3-EAAT2-tdT mice, eGFP is expressed in all cortical astrocytes and tdT is only expressed in a subset of the eGFP^+^ cells (28%) [12]. Using astrocytes prepared from the cortices of young (PND1-3) mice [21], eGFP expression has been used to study transcriptional regulation of GLT1 [16, 17, 56, 57]. Several members of the Robinson laboratory have been unable to induce tdT expression in these same cultures. This prompted us to determine where tdT is expressed early in development. In coronal sections of PND1 mice expressing both transgenes, we find that eGFP^+^ cells are widely dispersed throughout the brain, including both cortical and subcortical regions (Fig. 1a). Based on their morphology, these eGFP^+^ cells are likely immature astrocytes (Fig. 1b). In contrast to the widespread expression of eGFP, tdT^+^ cells are restricted to the striatum, subventricular zone, and white matter tracts in the ventral hindbrain at PND1 (Fig. 1c and d). At higher magnification, no tdT^+^ cells are observed in the cortex at PND1 (Fig. 1b, upper panel), while eGFP is easily detected. Unlike that observed in the more mature nervous system, tdT was never observed in any cortical or SVZ cells that expressed eGFP at PND1.

As indicated in the introduction, many cortical astroglia are generated from progenitors that originate in the ventricular and/or subventricular zone (VZ and SVZ) [22, 29, 30, 58–60]. We find that, as is observed for radial glia, eGFP^+^ cells are directly adjacent to the lateral ventricle (LV), while the tdT^+^ cells are positioned in the subventricular zone. Unlike eGFP^+^ cells, we never observed tdT^+^ processes in contact with the ventricle (Fig. 1b, lower panel). This suggests that tdT is not expressed in radial glia. A zoom-in of the SVZ in a sagittal section shows that tdT^+^ cells have a simple morphology, with only one or two processes that extend toward the cortex and resemble classical progenitor cells (Fig. 1d). All tdT^+^ cells visualized at PND1 contained one or two processes only (observed by at least three different researchers).

Prior studies have focused on analyses of 8.3-EAAT2-tdT in PND21 or older mice [12–14]. In the present study, we examined the expression of both reporter proteins in double reporter mice throughout development, focusing on the ventricular zone and cortex. At PND1, no tdT^+^ cells are found in the cortex while eGFP^+^ cells are relatively abundant (Fig. 1e, 1st panel). At PND 7, few tdT^+^ cells were found in the cortex and are abundant in the SVZ (Fig. 1e, 2nd panel). Over the next 7 days, the numbers of tdT^+^ cells increase in the cortex, and by PND14 there are no tdT + cells in the SVZ (Fig. 1e, 3rd & 4th panels). Once tdT^+^ cells are found in the cortex (starting at PND7), tdT overlaps with eGFP (Fig. 1f). We found that the number of tdT^+^ cells in the cortex is higher at PND21 than in older (PND60-90) mice (Fig. 1e). This is consistent with earlier studies that, although not examined in parallel, have documented higher numbers of tdT^+^ expression in cortical astrocytes in juvenile mice, PND25, up to 80% [15] than in adult mice (PND 60–90) where only 28% of the cortical astrocytes express tdT [12] (Fig. 1e, 5th panel).

Prior studies have demonstrated that GLT-1 protein expression increases during development, peaking during synaptogenesis [2, 61]. Using identical microscope settings, eGFP expression follows a similar time course with much lower levels at PND1 and PND7 (Fig. 1f). Although we were a bit surprised by the high levels of both reporters in SVZ at PND1, almost 30 years ago, two different groups demonstrated that GLT1 mRNA levels are quite high in the SVZ and olfactory bulb at PND1 [62, 63] (Online Resource 1, Panel a, reproduced with permission [62]). Similarly, the expression of GLT1 protein by immunohistochemistry on PND1 rat sagittal sections is higher in these two regions, although it is more widely distributed throughout the brain (Online Resource 1, Panel b, copyright 1997 Society for Neuroscience [2]). We examined GLT1 immunoreactivity and compared its distribution to eGFP and tdT. While eGFP expression largely overlaps with GLT1 immunoreactivity (Online Resource 1 Panel c), tdT expression is mainly found in the SVZ, striatum, and a small region in the posterior brain; therefore, the pattern of tdT expression more closely overlaps that of GLT1 mRNA (see Online Resource 1 Panel d vs Panel a).

### Migration of tdT^+^ Positive Cells in Organotypic Slice Cultures

**Fig. 2:**
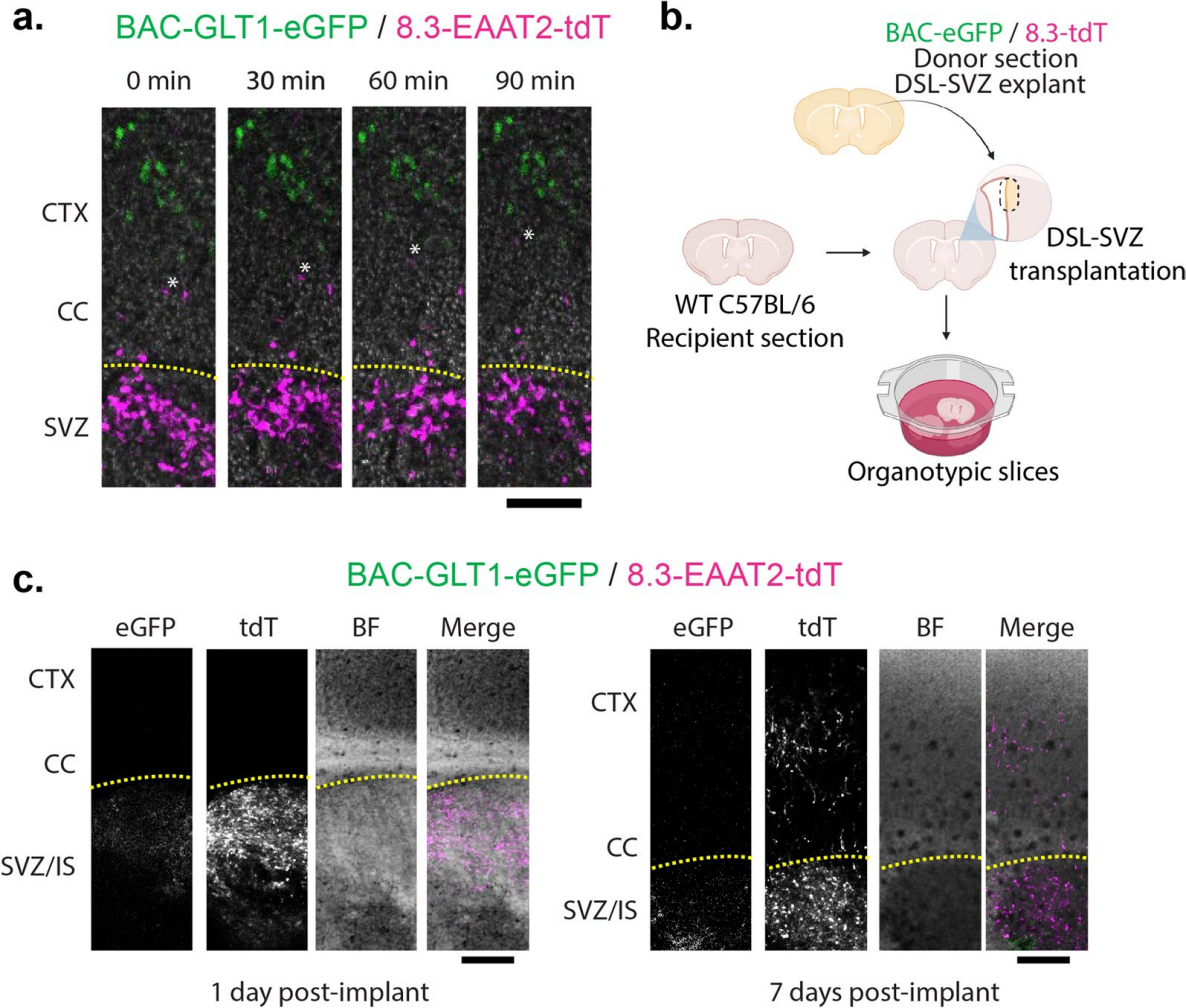
tdT^+^ cells migrate postnatally, while the eGFP^+^ cells remain stationary. **a** Organotypic brain slices were prepared from BAC-GLT1-eGFP/8.3-EAAT2-tdT PND1 mice. After a one-hour recovery period, the slices were transferred to a confocal microscope and sequential images of the SVZ region were taken every thirty minutes. Scale bar = 100 µm, CTX = cortex, CC = corpus callosum, SVZ = subventricular zone. *Highlights a migrating tdT^+^ cell, yellow dash line indicates the outer edge of the SVZ, n = 4. **b** Brains were collected from PND 1 to 2 pups of litters of double reporter BAC-GLT1-eGFP/8.3-EAAT2-tdT crossed with wild-type (WT) mice. The brains were genotyped on an epifluorescence microscope. Organotypic brain sections were prepared from double positive (BAC-GLT1-eGFP/8.3-EAAT2-tdT) and WT brains from littermate pups. Sections from WT (double negative) brains were used as hosts/recipients. 1 mm diameter pieces of the DSL-SVZ of the BAC-GLT1-eGFP/8.3-EAAT2-tdT (double positive) brain sections were used as implants and transplanted into the WT-host sections. **c** The implanted organotypic brain sections were grown in vitro and imaged in a confocal microscope 1 day or 7 days post-implant. BF = bright field, IS = implant site. Scale bars = 200 µm, n = 8

The fact that eGFP^+^ cells appear in the cortex before double-positive cells suggests that double-positive cells may migrate into the cortex later than eGFP^+^ cells. To test this possibility, we prepared coronal slices of PND1 double reporter mice and imaged the same fields every 30 min for up to 2 h. While eGFP^+^ cells remained in the same position throughout the experiments, tdT^+^ were observed migrating from the SVZ towards the cortex (Fig. 2a). We calculated the speed at which tdT^+^ cells migrated toward the cortex; the average speed was 39 ± 10 µm/h (5 cells/slice, 2–3 slices per mouse, 3 independent experiments), consistent with the rate reported elsewhere for astrocyte precursor cell migration [28, 35, 58, 64, 65]. To rule out the possibility that eGFP^+^ cells latently express tdT after migration into the cortex, we prepared slices from double negative (WT, recipient) and double positive (eGFP^+^/tdT^+^, donor) PND1 littermates. We transplanted a 1 mm diameter circular punch of the DSL-SVZ from the double reporter to the wild-type tissue using a method adapted from [66] (Fig. 1b). These slices were maintained in an incubator and imaged 1, 3, 5, and 7 days post-implant.

At one day post-implant, both eGFP^+^ and tdT^+^ cells remain in the implant site (Fig. 2c, left panel). At three days post-implant, the majority of the tdT^+^ cells that left the implant site were found in the CC, while at five days post-implant, the tdT^+^ cells were distributed laterally along the CC, with a few tdT^+^ cells appearing in the cortex. At seven days post-implant, tdT^+^ cells are found throughout the cortex (Fig. 2c, right panel). Although we cannot rule out the possibility that tdT^−^ cells could also be migrating out of the implant site and expressing tdT after migration, these observations strongly suggest that at least some of the tdT^+^ cells exit the DSL-SVZ, migrate laterally in the CC, and then migrate radially in the cortex. Similar results were observed using explants from single reporter mice; at 1- or 7-days post-implant, essentially no (1–2) eGFP^+^ cells are found outside the implant, while tdT^+^ are found throughout the cortex (Online Resource 2). When we implanted a 1 mm diameter circular punch of the striatum from the double reporter into the DSL-SVZ of the wild-type tissue, no migration was observed (n = 3, data not shown). This data suggests that something intrinsic to the DSL-SVZ tdT^+^ cells induces their migration and that not all tdT^+^ cells are alike.

### scRNAseq of eGFP^+^ and tdT^+^ Cells at PND1

**Fig. 3:**
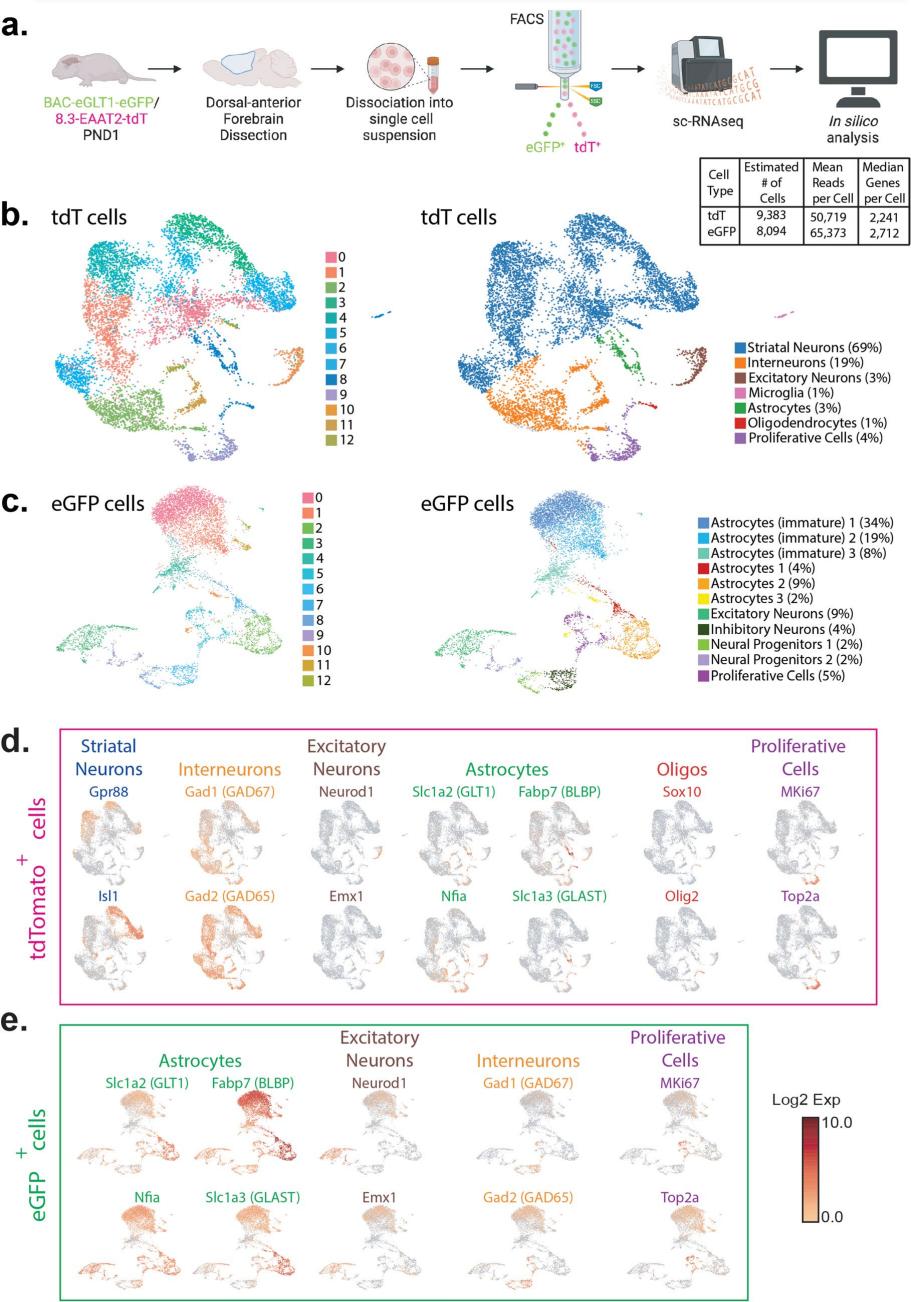
The tdT^+^ cells are a heterogeneous population of cells. **a** The dorsal-anterior forebrain of double reporter BAC-GLT1-eGFP/8.3-EAAT2-tdT PND1 mice was dissected and dissociated into a single-cell suspension. This suspension was subjected to fluorescence-activated cell sorting (FACS) to separate the eGFP^+^ and the tdT^+^ cells, followed by single cell-RNA sequencing (sc-RNAseq). The table indicates the number of cells analyzed and other quality control information. **b** & **c** UMAP-plot of sc-RNAseq tdT^+^ (Panel **b**) or eGFP^+^ (Panel **c**) cells with their cell type classification-based expression of canonical cell type markers as assigned by Loo and collaborators PND 0 mice [67]. **d **& **e** Canonical markers for the different cell types expressed in the corresponding region of the UMAP-plots. Color scale indicates the level of expression

**Fig. 4:**
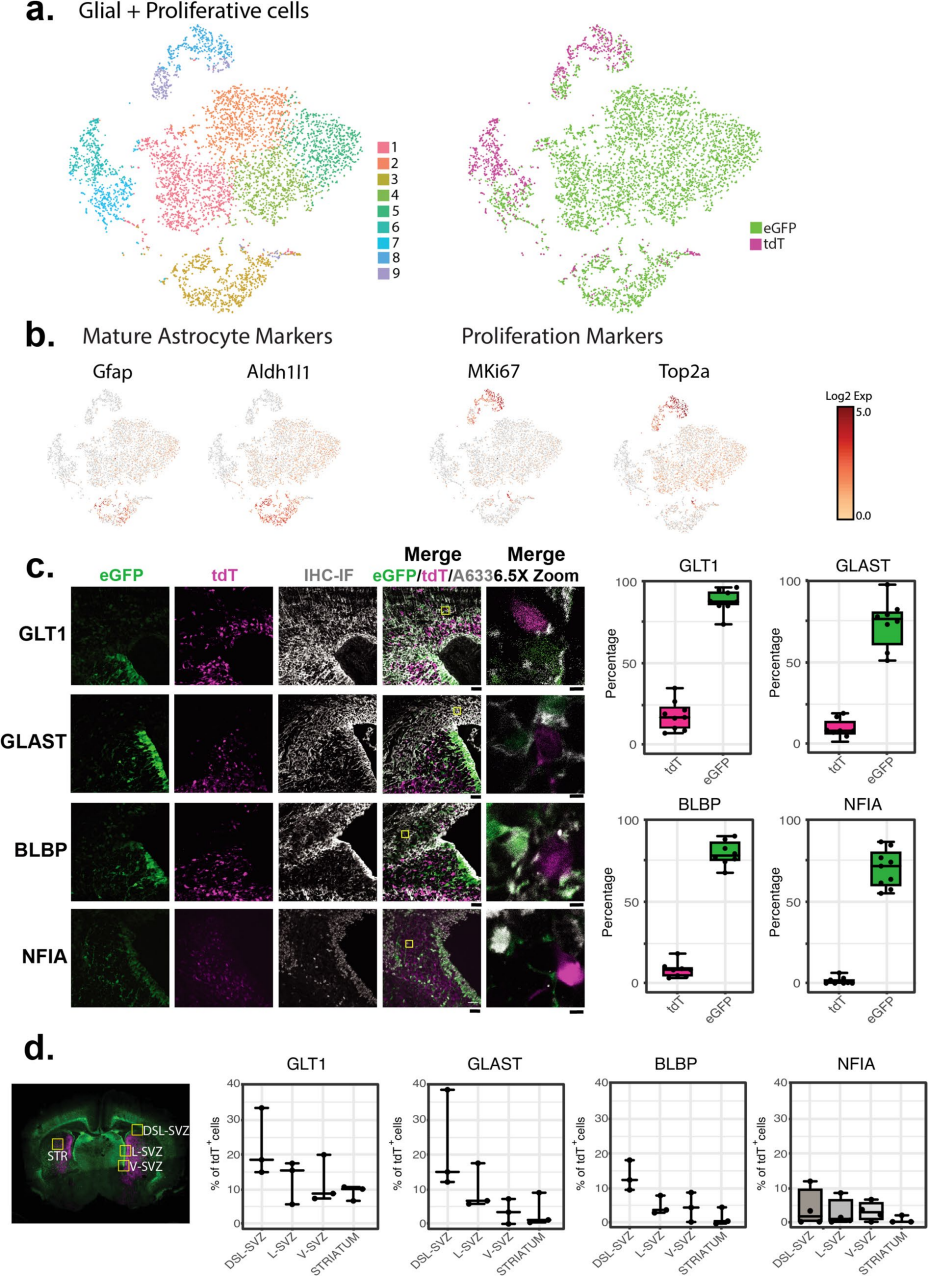
A subpopulation of tdT^+^ cells express astrocyte markers. **a** UMAP-plots of the tdT^+^ and eGFP^+^ glial and proliferative cells highlighted in Online Resource 3. **b** Canonical markers for mature astrocytes or proliferative cells in the corresponding regions of the UMAP-plots. Color scale indicates the level of expression. **c** IF was performed in double reporter BAC-GLT1-eGFP/8.3-EAAT2-tdT PND1 mice for the following astrocyte markers: GLT1, GLAST, BLBP, and NFIA. The dorsolateral (DSL), lateral (L), and ventral (V) regions of the subventricular zone (SVZ) and the striatum were imaged. Representative images from the DSL-SVZ region are shown. Scale bars for the first four columns = 50 µm. The fifth column shows higher magnification images of the regions indicated in the yellow boxes of the images in the fourth column. Scale bars = 5 µm. The boxplots in panel **c** indicate the percentage of tdT^+^ or eGFP^+^ cells that express the respective astrocyte marker in the SVZ. n = 9. **d** Coronal section indicating the different regions in which the expression of the astrocyte markers was analyzed. Graphs on the right represent the percentage of the tdT^+^ cells expressing GLT1, GLAST, BLBP, or NFIA in each brain region analyzed. n = 3–4

We were interested in understanding the molecular heterogeneity of the eGFP- and tdT-expressing cells at PND1. We dissected the dorsal-anterior forebrain, including the dorsal striatum, dorsal SVZ, and cortex, from double reporter mice and performed sc-RNAseq after FACS (Fig. 3a). As was observed by comparing the distribution of tdT and eGFP fluorescence (Fig. 1), fewer than 1% of cells expressed both reporters at PND1. These were excluded from sequencing. Over 8 thousand cells of each type were sequenced, with over 50,000 sequences identified per cell and over 2200 genes identified per cell. We performed QC, normalization, and dimensional reduction, followed by clustering in Seurat [42] with a 0.2 resolution. With this approach, 13 clusters of tdT^+^ cells and 13 clusters of eGFP^+^ cells were identified (Fig. 3b & c). Supplementary Tables 1 and 2 summarize the fold-differences (log2 scale) in mRNA levels observed in any one cluster compared to all other clusters in tdT^+^ cells and eGFP^+^ cells, respectively. The fraction of cells in the cluster that express a particular gene and the fraction of cells in all the other clusters that express the same gene are also presented with adjusted p values. The expression profiles of each cluster were compared to those of cell types previously identified in P0 mice [67]. Among the tdT^+^ cells, 69% were identified as striatal neurons, 19% interneurons, 3% excitatory neurons, 1% microglia, 1% oligodendrocytes, 4% proliferative cells, and 3% astrocytes (Fig. 3b, Supplementary Table 1). In contrast, eGFP^+^ cells clustered into 13 groups, of which 76% were astrocytes (61% immature and 15% mature), 9% excitatory neurons, 4% inhibitory neurons, 4% neural progenitors, and 5% proliferative cells (Fig. 3c, Supplementary Table 2). These data indicate that only a small fraction of tdT^+^ cells are in the astrocytic lineage, while three-quarters of the eGFP^+^ cells are astrocytic. Figures 3d & e show the relative expression patterns of known markers of each cell population. Comparison of the images in Fig. 3b and d allow one to see that cells identified as striatal interneurons, blue dots in 3b, express higher levels of Gpr88 and Isl1 [68–70]. Accordingly, others have shown that Gpr88 and Isl1 are almost exclusively expressed in the striatum (Online Resource 3a and b). These striatal interneurons also express higher levels of other markers of interneurons (Gad1 and Gad 2) (Clusters 0,1, and 3–7, Fig. 3b and Supplementary Table 1). There is also another population of cells that were identified as interneurons (express Gad1 and Gad2), but do not express the striatum markers (orange dots in 3b) (Clusters 2, 11, and 12, Fig. 3b and Supplementary Table 1). Similarly, cells identified at excitatory neurons express higher levels of Neurod1 and/or Emx1 [71–73] (Brown dots, cluster 10 in Fig. 3b and Supplementary Table 1). Meanwhile, astrocytes express higher levels of known markers of astrocytes, Slc1a2, Fabp7, Nf1a, and/or Slc1a3 [3, 7, 74–81] (Green dots, Cluster 8 in Fig. 3b and Supplementary Table 1). Oligodendrocytes express higher levels of known markers of this cell type, Sox10 and/or Olig2 [82–84] (Red dots, cluster 8 in Fig. 3b and Supplementary Table 1), and proliferative cells express high levels of the proliferation markers MKi67 and/or Top2a [85–87] (Purple dots, cluster 9 in Fig. 3b and Supplementary Table 1). The same markers were used to classify the eGFP^+^ cells (Fig. 3c and e, Supplementary Table 2). We integrated the datasets from tdT^+^ and eGFP^+^ cells using the Harmony algorithm to identify transcriptional differences [43]. The integrated clustering of the tdT^+^ and eGFP^+^ cells resulted in 16 clusters, with the tdT^+^ cells and the eGFP^+^ cells showing population separation in the resultant uniform manifold approximation and projection for dimension reduction (UMAP) plot (Online Resource 3c and 3d). We were interested in comparing the transcriptomes of the tdT^+^ and eGFP^+^ glial cells (astrocyte precursor cells, astrocytes, oligodendrocytes) and proliferative cells (outlined with a dashed black line on Online Resource 3e). These cells were re-clustered into 9 clusters (Fig. 4a), and 5 contained tdT^+^ cells. Markers of mature astrocytes (Gfap and Aldh1L1) were only found in clusters that exclusively contain eGFP^+^ cells. In contrast, markers of proliferative cells (Ex: MKi67 and Top2a) were only found in clusters that exclusively contain tdT^+^ cells (Fig. 4b). These data suggest that tdT^+^ cells are more immature than eGFP^+^ cells.

We examined the percentage of tdT^+^ or eGFP^+^ cells in the SVZ that express immunoreactivity for the glial glutamate transporters, GLT1 (gene Slc1a2) and GLAST (gene Slc1a3), or for immature astrocyte markers, basic lipid binding protein (BLBP) (gene Fabp7) and nuclear factor 1-A (NFIA) (gene Nfia). Only 15 ± 9% of tdT^+^ positive cells expressed detectable levels of GLT1 protein, 7 ± 6% of expressed GLAST, 8 ± 5% expressed BLBP, and 0.4 ± 2% expressed NF1A (Fig. 4c). In comparison, 87 ± 7 of eGFP^+^ cells expressed GLT1, 76 ± 15% expressed GLAST, 79 ± 8 express BLBP, and 72 ± 11 express NF1A. These data are consistent with the notion that most eGFP^+^ cells are astrocytes while the tdT^+^ cells are heterogeneous even within the SVZ. A study by Tsai and colleagues showed that the origin of different astrocyte subpopulations can be traced back to dorsal, lateral, or ventral regional domains [88]. To determine if the percentage of tdT^+^ cells that express glial markers varies in these domains, we quantified co-localization of GLT1, GLAST, BLBP, or NF1A with tdT in dorsal, lateral, and ventral SVZ (Fig. 4d). In these analyses (n = 3–4), the % of cells do not vary dramatically in these different regional domains. In the striatum, fewer than 10% of cells expressed these markers.

### Characterization of tdT^+^ Cells 7 Days After Explant

**Fig. 5:**
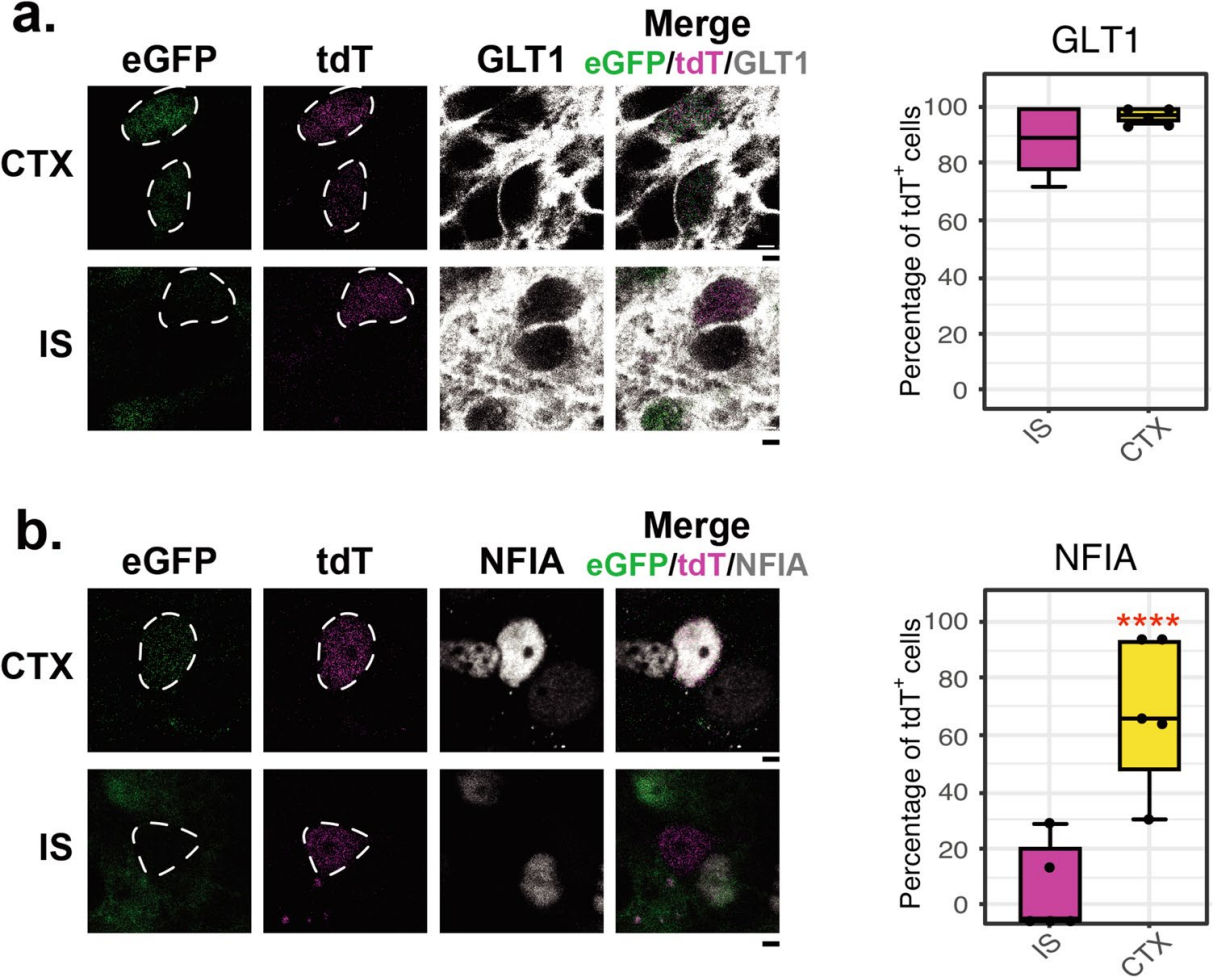
The tdT^+^ cells express eGFP, NFIA, and eGFP after migration into the cortex. Implanted organotypic brain sections were prepared as described in Fig. 2b and were grown in vitro for seven days. After this period, the sections were fixed, and IF was performed against GLT1 (Panel **a**) or NFIA (Panel **b**). Images were taken using a confocal microscope in a sequential mode to avoid bleeding from other channels in the implant site (IS) (bottom images), and the cortex (CTX) (top images). Boxplots show the percentage of the tdT^+^ cells that expressed GLT1 or NFIA. n = 4, t-test ****p < 0.001, representative images are shown, scale bars = 50 µm

This relatively low percentage of tdT^+^ positive cells that express GLT1, GLAST, BLBP, or NF1A even in DSL-SVZ at PND1 prompted us to examine the expression of these markers in tdT^+^ after they migrate into the cortex in organotypic slices. We quantified the % of tdT^+^ cells that express GLT1 or NF1A in the implanted tissue and cortex after seven days in culture. Most tdT^+^ cells in the implant site (IS) or the cortex expressed GLT1 (89 and 97% respectively) (Fig. 5a). Only 11% of tdT^+^ cells in the implant express NF1A, while just over 75% of tdT^+^ cells in the cortex express NF1A (76%) (Fig. 5b). No tdT^+^ cells in the implant also express eGFP, but 100% of cells that migrate into the cortex express eGFP after 7 days (Fig. 5 a & b). These studies suggest that the non-glial cells in the implant die over seven days, that a large percentage of these cells are in the glial lineage even though only a small percentage of these cells express GLT1, GLAST, BLBP, or NF1A at PND1 or that these cells stop expressing tdT as they mature.

### Effects of iGluR Antagonists or a Pan Glutamate Transport Inhibitor on the Migration of tdT^+^ Cells

**Fig. 6:**
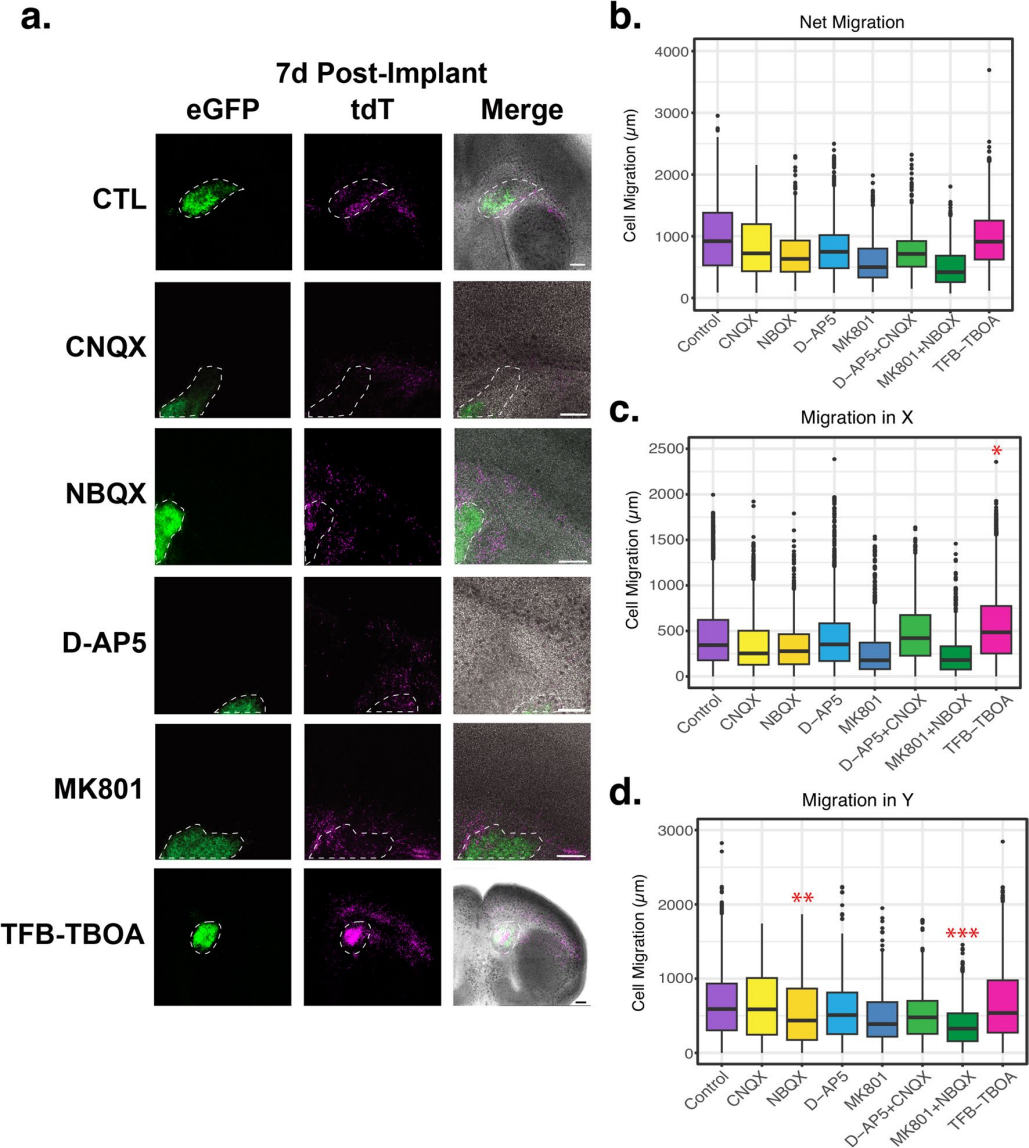
Glutamate signaling regulates the migration of the tdT^+^ cells. **a** Implanted organotypic brain sections were prepared as described in Fig. 2b, and after a one-hour recovery period, they were cultured in the presence or absence of antagonists of the ionotropic glutamate transporters or a blocker of the glial glutamate transporters. Seven days later, they were imaged. n > 4, representative images are shown. Scale bars = 250 µm. The dashed lines indicate the implant site. **b**, **c**, & **d** The boxplots show the net migration of the tdT^+^ cells out of the implant site (Panel **b**), or their migration from the implant site in the direction of the X-axis (lateral migration) (Panel **c**) and the Y-axis (radial migration) (Panel **d**). Colors indicate families of receptor antagonists i.e. AMPAR and KAR (yellow), NMDAR (blue) combination of the AMPAR, KAR, and NMDAR (green), and the glutamate transporter blocker TFB-TBOA (pink). n > 4, *p < 0.05, **p < 0.01, ***p < 0.005 for comparison with control

Diverse molecular signals direct the migration of neuronal progenitors into the cortex, including chemotactic molecules like netrins, semaphorins, and slits; secreted factors and neurotransmitters like brain-derived neurotrophic factor, neurotrophin 4, GABA, and glutamate; and matrix- and membrane-bound molecules like Reelin, EphB2, and heparin sulfate glycosaminoglycans (Reviewed in [89–92]). Blocking NMDA receptors decreases granule cell migration while either blocking glutamate transport or enhancing NMDA receptor activation increases the migration of these neuronal progenitors [36]. Glutamate signaling has also been shown to increase the rate of migration of cortical neurons from the germinal zones to the cortex [37–40], GABAergic interneurons [41], and oligodendrocyte precursor cells [93]. On the other hand, relatively few studies have examined the control of migration of glial progenitors into the cortex, in part, because it has been difficult to mark these cells optically. We determined if glutamate signaling regulates the migration of tdT^+^ cells into the cortex. To do this, we implanted a 1 mm diameter section of the DSL-SVZ area of double reporter mice into the DSL-SVZ of WT organotypic brain sections (Fig. 2b). We grew them in vitro for 7 days in the presence or absence of ionotropic glutamate receptor (iGluRs) antagonists. For NMDA receptors, we used 10 µM MK801 or 100 µM D-AP5, and for non-NMDA (AMPA and KA) receptors, we used 10 µM NBQX or 30 µM CNQX. Because there is some controversy over the type of glutamate receptors expressed in astrocytes in development, and to evaluate whether both types of receptors regulate the tdT^+^ cells migration, we also used two different combinations of NMDA and non-NMDA receptor blockers, MK801 + NBQX or D-AP5 + CNQX. Blocking glutamate transport both causes an increase in extracellular glutamate and prevents signaling that can occur through the transporters themselves (for a review, see [6]). We used a pan blocker of the glutamate transporters GLT1, GLAST, and EAAT3, 2 µM TFB-TBOA. In Fig. 6a and Online Resource 6a, we show representative images of the localization of eGFP^+^ and tdT^+^ cells grown in the presence of the iGluRs antagonists or TFB-TBOA 7 days post-implant; the implant site is outlined with dashed lines. Seven days post-implant, no significant effect was observed in the net migration of tdT^+^ cells (Fig. 6b). It has been reported that astrocyte precursor cells leave the germinal zone by erratic migration, followed by a radial/blood vessel-guided migration [28, 35, 94]. Therefore, we analyzed the effects of iGluR antagonists and the blocker of the glutamate transporters, measuring the migration in the x direction (lateral migration) and y direction (radial migration). Compared to control, only the blocker of the glial glutamate transporters, TFB-TBOA, altered the lateral migration (X-axis) of tdT^+^ cells (Fig. 6c); it increased the lateral migration of the tdT^+^ cells to 162% of control (representing a 97 µm increase) (p = 0.029). Meanwhile, NBQX, an AMPA receptor antagonist, alone or in combination with MK801, a non-competitive NMDA receptor antagonist, decreased the radial migration (Y-axis) of the tdT^+^ cells by 40 to 50% (Fig. 6d, p = 0.010 & p = 0.009). We also calculated the radial migration of the tdT^+^ cells into the cortex by measuring the distance these cells migrated from the white matter tracts of the corpus callosum. We found that NBQX decreased the migration of the tdT^+^ cells by 50% (Online Resource 6b, 0.5-fold, p = 0.002).

**Fig. 7:**
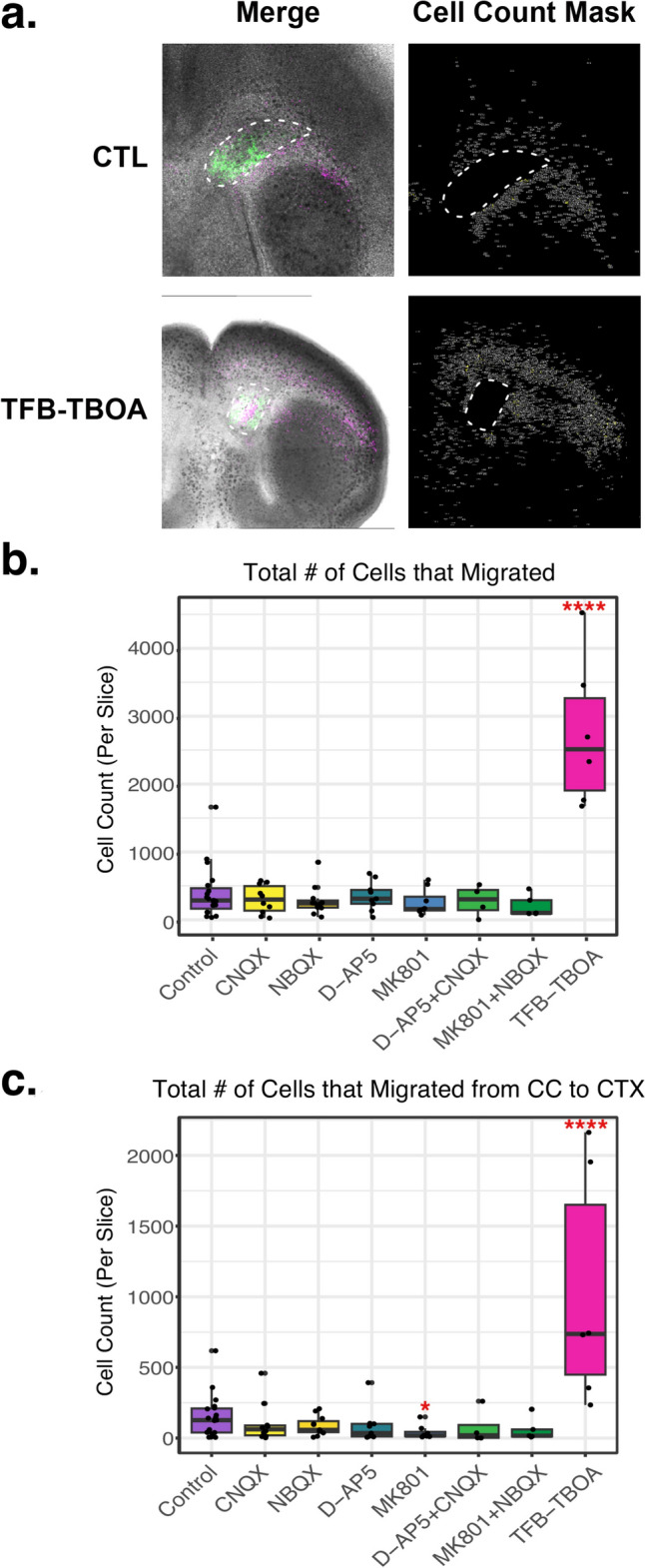
Inhibition of glutamate transporters increases the number of tdT^+^ cells that migrate. **a** Implanted organotypic brain sections were prepared as described in Fig. 2b. After a one-hour recovery period, they were cultured in the presence or absence of antagonists of the ionotropic glutamate transporters or a blocker of the glial glutamate transporters. Seven days later, they were imaged, and the number of tdT^+^ cells that migrated were quantified. n > 4, representative images for control (non-treated) and treatment with TFB-TBOA are shown. The right images show the masks generated in Fiji of the images shown on the left; these masks allowed for automatic quantification of the number of tdT^+^ cells that migrated. Dash lines indicate the implant site. **b** & **c** Boxplots show the quantification of the number of tdT^+^ cells that migrated out of the implant site (Panel **b**) and the number of tdT^+^ cells that migrated into the cortex (Panel **c**) normalized per brain section. Colors indicate families of receptor antagonists, i.e., AMPAR and KAR (yellow), NMDAR (blue), the combination of the AMPAR, KAR, and NMDAR (green), and the glutamate transporter blocker TFB-TBOA (pink). n > 4, *p < 0.05, ****p < 0.001 for comparison with control (purple)

We observed that TFB-TBOA not only increased the lateral migration of the tdT^+^ cells but also substantially increased the number of cells in the cortex (Figs. 6a and 7) (p < 0.001). In Fig. 7a, in the right column, we show representative images of the mask used to automatically quantify the tdT^+^ cells that migrated out of the implant site (outlined with dash lines). We observed a tenfold increase in the number of tdT^+^ cells found outside of the implant site after 7 days of treatment with TFB-TBOA (Fig. 7b, p < 0.001) and a sixfold increase in the number of tdT^+^ cells found in the cortex after the same treatment (Fig. 7c, p < 0.001). It is possible that TFB-TBOA is increasing the migration of tdT + cells, triggering proliferation of the precursors that migrate into the cortex, or it may be inducing expression of tdT in cells that don’t express tdT in the absence of TFB-TBOA. It will be important to differentiate between these possibilities in future studies.

## Discussion

In the present study, we characterized a double reporter mouse line (BAC-GLT1-eGFP/8.3-EAAT2-tdT) that uses the whole GLT1 mouse gene to control the expression of eGFP and 8.3 kb of the proximal region of the GLT1/EAAT2 human promoter to control expression of tdT. It had been reported that in adult mice, this double reporter mouse line allows for the visualization of a subpopulation of cortical astrocytes due to their unique expression of tdT (in double positive eGFP^+^/tdT^+^ cells). Unlike that observed in adult mice [12], we did not observe any double positive cells at PND1 in the cortex or SVZ, and FAC sorting showed that essentially no cells (less than 1% of > 650 K cells analyzed) express both proteins at this age. At PND1, eGFP is distributed throughout the brain, and in the cortex, it is found in immature astrocytes. Meanwhile, tdT is enriched in the SVZ, striatum, and white matter tracts in cells that resemble progenitor cells, due to their simple morphology with only one to two processes. At PND1, the tdT^+^ cells are actively migrating from the SVZ to the cortex, while the eGFP^+^ cells remain static throughout the experiments. Using scRNA-seq and IF, we found that the tdT^+^ cells are a heterogenous population of progenitor cells, while the majority of eGFP^+^ cells are already committed to the astrocyte lineage. We found that after migration and once in the cortex, the tdT^+^ cells acquire expression of eGFP and NFIA, an early astrocyte marker. We also found that an AMPA receptor antagonist decreases the radial migration of the tdT^+^ cells. Meanwhile, a blocker of the glutamate transporters GLT1 and GLAST increases the lateral migration of tdT^+^ cells and the number of these cells that migrated out of the implant by tenfold. This finding strongly supports the role of glutamate signaling in the migration of tdT^+^ astrocyte precursor cells and the number of these cells that end up in the cortex. However, our study has limitations: 1. Our system does not permanently label cells and, therefore, cannot be used for lineage tracing. In future studies, a permanent labeling system will be needed to dissect the migration patterns and to examine the contribution of these tdT^+^ progenitors to the cortical astrocyte population. In addition, it will be important to follow the migration of these cells using a time-lapse video tracking; this, combined with morphology analysis, will allow us to determine the migration pattern used by these astrocyte precursor cells. Despite these limitations, the data presented suggest that precursor cells give rise to astrocytes in different regional and temporal contexts. Here, we also show that environmental cues drive the migration and specification of astrocyte precursor cells.

### Expression of tdT in Interneurons

Using scRNA-seq, we found that tdT, driven by 8.3 kb of the human GLT1/EAAT2 promoter, is expressed in a heterogeneous population of cells early in development, including interneurons and striatal inhibitory neurons (Fig. 3). IHC-IF also shows that these cells are heterogeneous. The fact that no striatal cells migrate from explant cultures supports functional heterogeneity of these cells. Although GLT1 is expressed in oligodendrocyte precursor cells (OPCs) [11, 95–97] and in neurons [98–103] early in development, the fact that GLT-1 is not found in all of these cells strongly suggests that 8.3 kb of the human promoter is not sufficient to selectively control expression of tdT in GLT1-expressing cells. The proximal portion of the human promoter was to generate these mice; perhaps there are species differences in the sequences required for astrocyte specific-expression. It is also possible that 8.3 kb is not sufficient for cell-selective labelling. In fact, a similar lack of cell specificity has been observed when fragments of the mouse GFAP-promoter are used (Reviewed in [104–107]). In earlier studies, we mapped the evolutionarily conserved domains found in the mouse and human promoter; and there are six additional conserved domains of at least 100 nucleotides distal to the 8.3 kb [19].

### Generation of Astrocyte Heterogeneity

It had been shown that oligodendrocytes are generated by different waves of OPCs in a ventral to dorsal pattern in the spinal cord and forebrain (For a review see [108]). However, there is no evidence that these different OPC sources generate any functional heterogeneity. In fact, when OPCs from one of these sources are ablated, neighboring populations quickly expand and fill the ‘empty’ territory [109]. This has contributed to the hypothesis that OPCs are a homogenous population of cells early in development, and that oligodendrocytes and OPCs become heterogenous in the juvenile and adult brain thanks to the contribution of environmental signals [110, 111].

For astrocytes, there is evidence that neuronal [13, 18, 20, 112–117] and endothelial [17, 18, 118, 119] cues regulate the expression of genes critical for astrocyte functions, including GLT1, suggesting that the environment contributes to the specification of astrocytes. Accordingly, Farmer and collaborators demonstrated that neurons, through Shh signaling, regulate core properties of cerebellar astrocytes and Bergmann glial cells [120]. In the cortex, astrocytes are arranged in layers with unique gene expression patterns and distinct morphologies. Two independent groups demonstrated that when neuronal cortical layers are disrupted, astrocytes lose their layer-specific properties [121, 122]. This data strongly indicates that environmental cues drive astrocyte heterogeneity. However, other evidence also suggests that intrinsic properties (diversity of astrocyte precursor cell lineages) contribute to the astrocyte heterogeneity observed in adult animals. Hochstim and collaborators demonstrated the existence of three domains in the spinal cord that give rise to distinct astrocyte subtypes regulated by the transcription factors Pax6 and Nkx6.1 [123]. Ohayon and collaborators demonstrated that the transcription factor Olig2 segregates a subtype of spinal cord astrocytes early in development [124, 125]. Liu et al. also identified two subtypes of astrocyte precursor cells based on the expression of Sparc or Sparcl1 [29]. Tsai and collaborators showed that astrocytes are allocated to regional domains in the spinal cord and the brain, which can be traced back to dorsal, lateral, or ventral birth regions based on the expression of Emx1, Dbx, or Nkx2.1, respectively [88]. And in this case, ablation of astrocytes from one domain does not recruit astrocytes from a different domain, indicating that the regional identity of astrocytes is fixed [88]. Accordingly, here we show that the tdT^+^ astrocytes originate postnatally from SVZ progenitors, with no detected contribution of direct radial glia transformation. Altogether, this suggests that intrinsic properties contribute to astrocyte heterogeneity. When we characterized adult double reporter mice (BAC-GLT1-eGFP/8.3-EAAT2-tdT), we found that Norrin (gene name NDP), Kir4.1 (gene name Kcnj10), Lgr6, and Olig2 were enriched in this astrocyte subpopulation (8.3-astroglia). Others have shown that expression of Norrin and Kir4 increases with age, picking up in adult mice [12, 126–129]. Accordingly, we did not detect Norrin nor Lgr6 in our scRNAseq analysis at PND1. Meanwhile, Kcnj10 and Olig2 were slightly enriched in the astrocyte population from the tdT^+^ cells (cluster 8 of Fig. 3b and Supplementary Table 1, 1.41 and 2.19 fold difference, respectively). However, no differential expression of these genes was found in the glial tdT^+^ cells versus the glial eGFP^+^ cells (Fig. 4a). Therefore, we could not determine if the migrating tdT^+^ cells generate the subpopulation of cells identified in our earlier study. This is one of the limitations of our study even though we performed scRNA-seq and IHC-IF, we could not identify specific markers for this subpopulation of astrocyte precursors. Likely because precursor cells have open and dynamic transcriptomes [130, 131], and because they represent a small population (~ 9% of the tdT + cells). Future studies in which this population is enriched before double-labeling or in which the scRNA-seq analysis is extended across different developmental stages would allow for further characterization of the tdT + astrocyte precursor cells and plausible for identification of stage-specific markers—followed by validation in wild-type mice, as was done in adult mice [12].

Parallel to OPCs, there is strong evidence that environmental cues further contribute to the generation of astrocyte heterogeneity (reviewed in [132]). Accordingly, we found that tdT^+^ precursor cells acquire the expression of eGFP and NFIA after they arrive at the cortex (Fig. 5) and that environmental cues (e.g., glutamate) regulate the migration of these cells (Figs. 6 and 7). Therefore, a complex interplay between intrinsic and extrinsic signals is responsible for inter- and intra-regional astrocyte heterogeneity.

### Migration of Astrocyte Precursor Cells: Glutamate-Dependent Mechanism

Although good advances have been made concerning the molecular signals that drive the migration of neuronal progenitors (reviewed in [32, 39, 133]), there are few studies on the migration of glial cells. It has been reported that glutamate acts as a chemoattractant that stimulates the radial migration of cerebellar granule cells [36] and cortical neuronal precursor cells [40] through NMDA receptor- and Ca^2+^-dependent mechanisms [36, 40, 134]. Glutamate has also been found to stimulate the migration of oligodendrocyte precursor cells [93]. In addition, we detected high levels of the glutamate transporter GLT1 in the SVZ at PND1, indicating important glutamate activity in that area. Therefore, we evaluated whether glutamate was involved in the migration of the tdT^+^ cells. Our results suggest that glutamate regulates the migration of the tdT^+^ SVZ progenitor cells from their birthplace to the cortex, where they differentiate into astrocytes, or that blocking glutamate transport induces the proliferation of the tdT^+^ cells. Both of these plausible explanations are consistent with the fact that mice deleted of both GLAST and GLT1 display multiple brain defects, including cortical malformation [102]. Further studies are required to dissect the role of glutamate in the regulation of the number of tdT^+^ cells that end in the cortex.

Glial progenitors migrate in an undirected/erratic manner in the SVZ and then follow radial and tangential paths guided, at least in part, by blood vessels to enter the white matter, cortex, and striatum [35, 65]. This is consistent with the pattern of tdT^+^ cell migration that we observed (data not shown). We evaluated radial and lateral migration, and interestingly, we found that blocking glutamate transporters with TFB-TBOA increases to 162% of control the lateral (almost 1 mm increase on migration), but not the radial, migration of the tdT^+^ cells (Fig. 6a and c). TFB-TBOA blocks the glutamate transporters GLT1, GLAST, and EAAC1 (EAAT1-3) with similar affinity [135]; therefore, further studies are required to explore the contribution of each glutamate transporter subtype. Alternatively, the effects of TFB-TBOA might be mediated by glutamate receptors. By blocking glutamate transporters, the glutamate extracellular concentration increases, activating glutamate receptors. Meanwhile, we found that NBQX, a blocker of AMPA and KA receptors, decreases by 50% the radial, but not the lateral, migration of the tdT^+^ cells (Fig. 6a and d, and Fig. 7b). These results suggest that glutamate regulates erratic and blood vessel-guided migration, but by different mechanisms with the former mediated by glutamate transporters and the latter by glutamate receptors. It is also not known whether glutamate regulates the migration of all astrocyte precursor cells (those derived directly from radial glia and/or only from SVZ progenitors) and whether their migration is regulated by different environmental cues depending on region and/or time of birth.

In this study, we tested the participation of different iGluRs by using different blockers. For AMPA receptors, we used CNQX or NBQX. We found that NBQX, but not CNQX, decreased the radial migration of tdT^+^ cells (Fig. 6d and Online Resource 6). Although we initially expected the same blocking effect, closer literature inspection suggests that CNQX can drive some AMPA current since it can be converted from a blocker to a partial agonist [61, 136, 137]. Meanwhile, NBQX effectively blocks the currents mediated by AMPAR at the concentrations used [138, 139]. This could indicate that even a small current through AMPAR is sufficient to induce the migration of tdT^+^ cells. We evaluated the expression of different AMPAR subunits in our scRNAseq data and found that Gria2, the GluR2 gene, is expressed in ~ 70% of the tdT^+^ cells. However, no differences in the expression of this or other AMPAR subunits were found in the different tdT clusters. Further studies are required to dissect the glutamatergic receptors involved in the migration of the tdT^+^ cells.

Another limitation of our study is that we used a highly unbalanced study design, with not all animals or slices receiving each pharmaceutical agent. Thus, the estimated treatment effects often combined a within- and between-animal comparison. Moreover, sample sizes differed somewhat depending on the pharmaceutical agent. While no power calculations were made in advance of the study, we anticipate that the detectable sample sizes are often large. For example, with a paired design (e.g., control versus treatment, each measured in the same animal), a sample of 3–5 animals has 80% power using a T-test to detect effect sizes (mean treatment effect/standard deviation) of 1.7–3.3. While our study detected what often appeared to be large effects, by graphically displaying all of the data we hope to provide information to readers who may wish to pursue further investigations into these phenomena.

### SVZ Progenitors Give Origin to Astrocytes

We are not the first to show that the neonatal SVZ generates cells that give origin to cortical astrocytes. A little over 30 years ago, the Goldman group demonstrated that astrocytes and oligodendrocytes develop from progenitors in the SVZ of postnatal rat forebrain. For this, they injected alkaline phosphatase- and beta-galactosidase-containing retroviruses in PND2-3 rats and harvested and imaged the brains 2- or 28-days post-injection [26]. In a follow-up study, they found that Olig2 is sufficient and necessary to prevent neuronal differentiation and to direct SVZ progenitors toward astrocytic and oligodendrocytic fates [140]. In this study, they also found that the expression of Olig2 is transient, with a gradual downregulation of Olig2 expression with age. In our study, we found that only 1% of the tdT^+^ cells express Olig2 (Fig. 3d), and that expression of Olig2 is found in a population of the proliferative cells (MKi67^+^ and Top2a^+^, Fig. 3d). Therefore, it is plausible that this 1% of the tdT^+^ cells were those identified by Goldman’s group. It is also plausible that more of the tdT^+^ cells are derived from the same population, but we cannot identify them due to the transient nature of Olig2 expression.

## Conclusion

In conclusion, our study found a subpopulation of cortical astrocytes generated from SVZ progenitor cells that migrate postnatally to the cortex. We found that glutamate signaling regulates the migration and number of these precursor cells and that once in the cortex, they gain expression of astrocyte markers. Our study shows that diverse astrocyte lineages contribute to the generation of astrocyte heterogeneity and that environmental cues regulate the migration and specification of astrocyte precursor cells.

## Supplementary Information

Below is the link to the electronic supplementary material.

Supplementary file1 (PPTX 4734 KB)

Supplementary file2 (XLSX 1178 KB)

Supplementary file3 (XLSX 2315 KB)

## Data Availability

The datasets generated during and/or analyzed during the current study are available in NCBI Gene Expression Omnibus website, GSE273223.
