# Supplementary material for: Identification of a Subpopulation of Astrocyte Progenitor Cells in the Neonatal Subventricular Zone: Evidence that Migration is Regulated by Glutamate Signaling
Source: Neurochem Res. 2025 Jan 9;50(1):77. doi: 10.1007/s11064-024-04326-2 (PMC11717811; doi:10.1007/s11064-024-04326-2)
Supplement: Supplementary file 1 — Supplementary file1 (PPTX 4734 KB) [file 11064_2024_4326_MOESM1_ESM.pptx]

## Slide 1
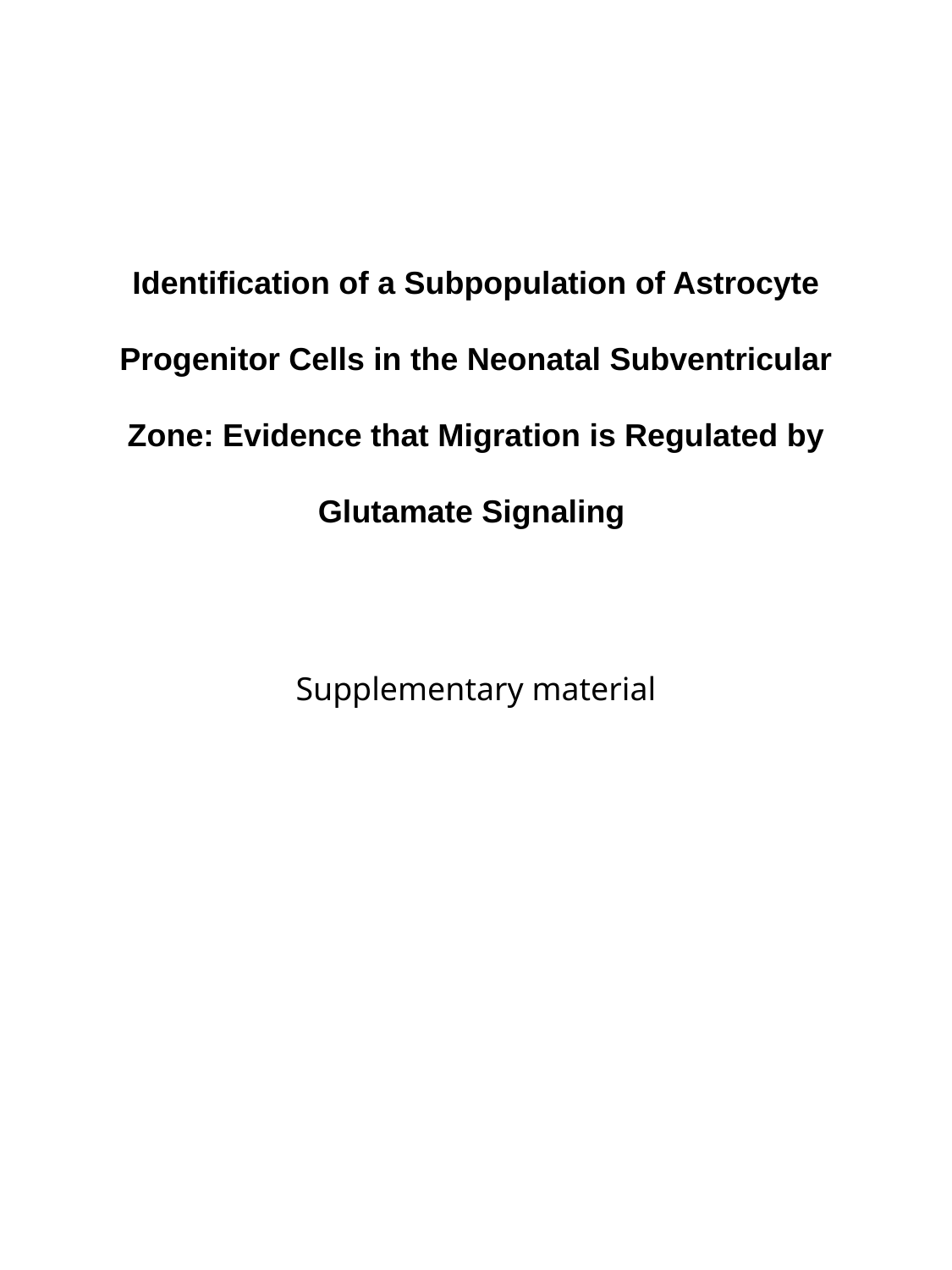

# Identification of a Subpopulation of Astrocyte Progenitor Cells in the Neonatal Subventricular Zone: Evidence that Migration is Regulated by Glutamate Signaling
Supplementary material

## Slide 2
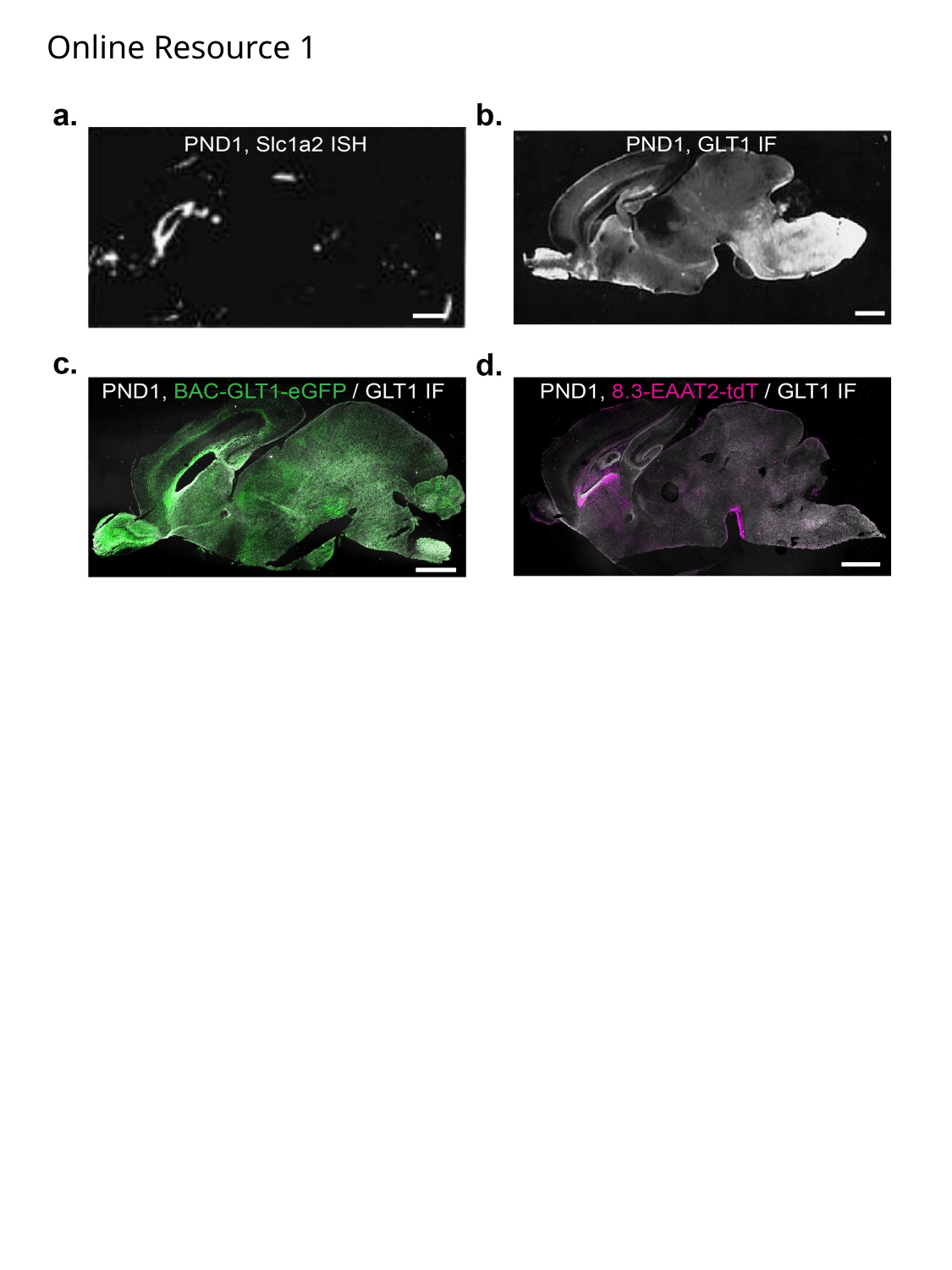

Online Resource 1

## Slide 3
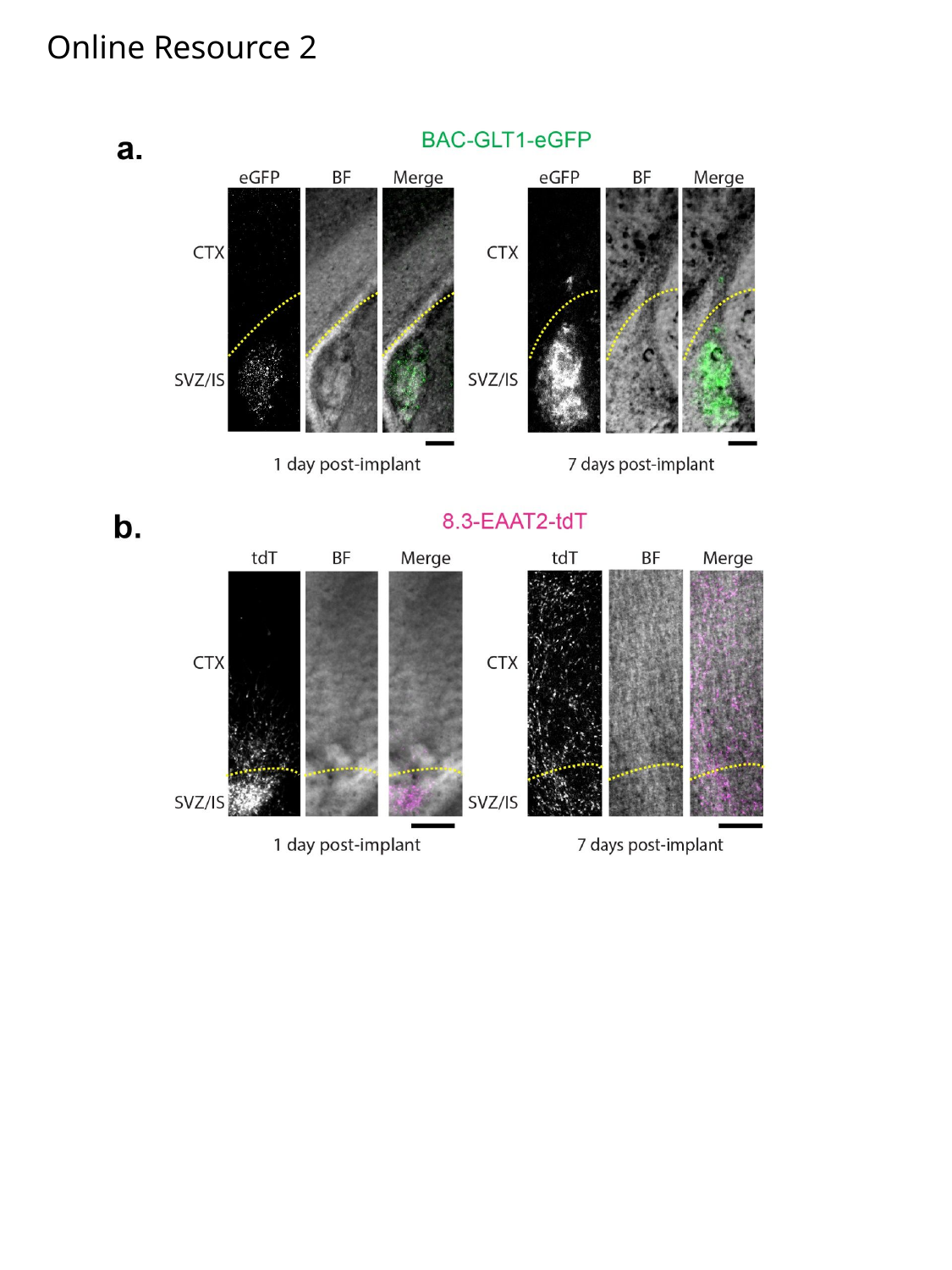

Online Resource 2

## Slide 4
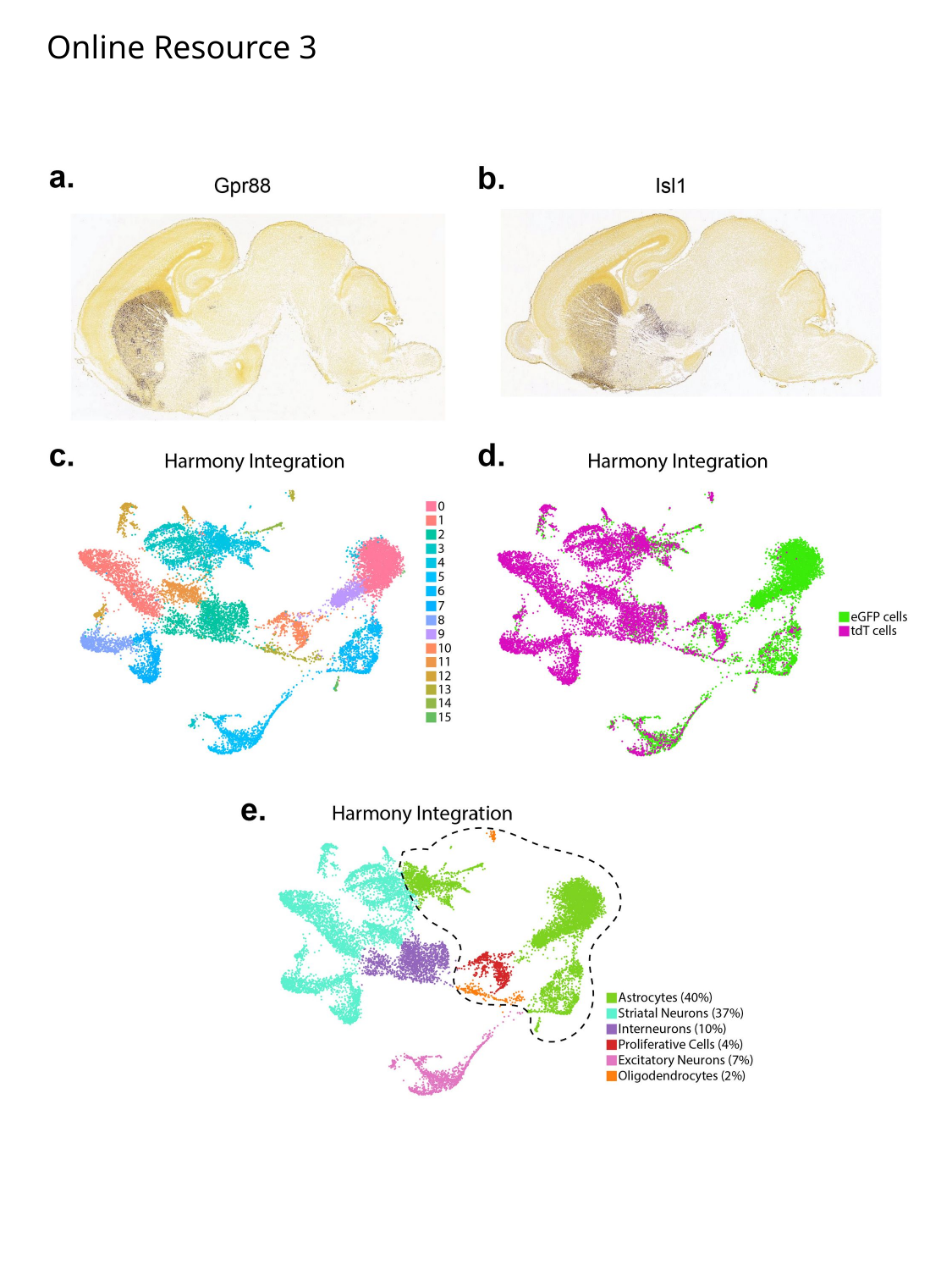

Online Resource 3

## Slide 5
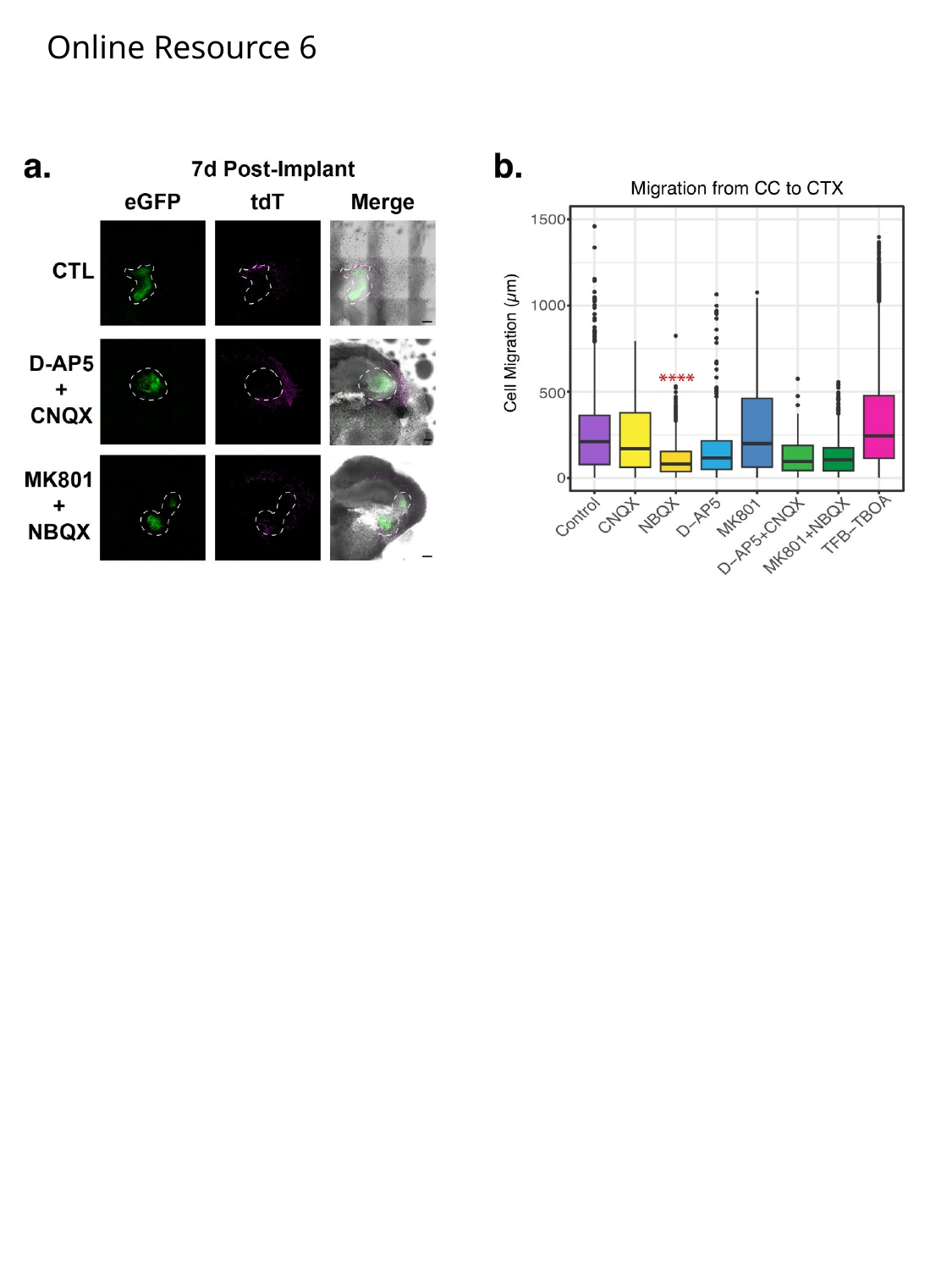

Online Resource 6
